# Wnt Signalling Pathway Parameters for Mammalian Cells

**DOI:** 10.1371/journal.pone.0031882

**Published:** 2012-02-21

**Authors:** Chin Wee Tan, Bruce S. Gardiner, Yumiko Hirokawa, Meredith J. Layton, David W. Smith, Antony W. Burgess

**Affiliations:** 1 Ludwig Institute for Cancer Research, Melbourne-Parkville Branch, Parkville, Victoria, Australia; 2 Faculty of Engineering, Computing and Mathematics, The University of Western Australia, Perth, Western Australia, Australia; 3 Melbourne School of Engineering, University of Melbourne, Parkville, Victoria, Australia; University of Washington, United States of America

## Abstract

Wnt/β-catenin signalling regulates cell fate, survival, proliferation and differentiation at many stages of mammalian development and pathology. Mutations of two key proteins in the pathway, APC and β-catenin, have been implicated in a range of cancers, including colorectal cancer. Activation of Wnt signalling has been associated with the stabilization and nuclear accumulation of β-catenin and consequential up-regulation of β-catenin/TCF gene transcription. In 2003, Lee et al. constructed a computational model of Wnt signalling supported by experimental data from analysis of time-dependent concentration of Wnt signalling proteins in Xenopus egg extracts. Subsequent studies have used the Xenopus quantitative data to infer Wnt pathway dynamics in other systems. As a basis for understanding Wnt signalling in mammalian cells, a confocal live cell imaging measurement technique is developed to measure the cell and nuclear volumes of MDCK, HEK293T cells and 3 human colorectal cancer cell lines and the concentrations of Wnt signalling proteins β-catenin, Axin, APC, GSK3β and E-cadherin. These parameters provide the basis for formulating Wnt signalling models for kidney/intestinal epithelial mammalian cells. There are significant differences in concentrations of key proteins between Xenopus extracts and mammalian whole cell lysates. Higher concentrations of Axin and lower concentrations of APC are present in mammalian cells. Axin concentrations are greater than APC in kidney epithelial cells, whereas in intestinal epithelial cells the APC concentration is higher than Axin. Computational simulations based on Lee's model, with this new data, suggest a need for a recalibration of the model.

A quantitative understanding of Wnt signalling in mammalian cells, in particular human colorectal cancers requires a detailed understanding of the concentrations of key protein complexes over time. Simulations of Wnt signalling in mammalian cells can be initiated with the parameters measured in this report.

## Introduction

Wnt signalling regulates survival, proliferation and differentiation at various stages of development [1], [2], [3], [4]. It has been proposed previously that a primary function of the Wnt pathway is to modulate the concentration of the multi-functional protein β-catenin [5], [6]. β-catenin has several known roles in cellular processes including cell adhesion, migration and transcription [7]. When the Wnt pathway is inactive, a β-catenin degradation complex is formed by the scaffold protein Axin and the multi-functional protein Adenomatous Polyposis Coli (APC) [8], [9]. This complex promotes the phosphorylation and degradation of β-catenin by glycogen synthase kinase-3-β (GSK3β) [10]. This phosphorylation targets the β-catenin for degradation via the proteasome [11]. According to current models [12], [13], [14]: upon activation of Wnt signalling, the degradation complex formation is disrupted, which leads to an increased concentration of β-catenin in the cell. A functional consequence of increased β-catenin appears to be an increased concentration of β-catenin:T-Cell Factor complexes in the nucleus and the activation of transcription of genes that promote cell proliferation [3], [15].

Interpreting the roles of particular signalling proteins is complex. For example, β-catenin is not only involved in gene transcription, but is also a key member of a cell-cell adhesion complex with E-cadherin [7], Axin also binds to many other different proteins in the cell, many of which are involved in both Wnt signalling [16] and in other signalling pathways such as TGFβ [17] and the JNK pathway [18]. APC is known to be a tumour suppressor protein, but it is also reported to be involved in cell adhesion [19], cell migration [20], cytoskeleton regulation [21] and chromosomal segregation [22].

The multifunctional nature of these proteins means that the Wnt signalling pathway also interacts with other major signalling pathways. Critically, the cellular adhesion pathway is tightly linked to the Wnt pathway - APC [23] and β-catenin [24] are involved in both pathways. Loss of cellular adhesion junctions is known to be one of the key hallmarks of cancer invasion and metastasis [25], [26], so it is unsurprising that mutations in β-catenin, Axin and APC have all been detected in human cancer [27]. In fact, abnormal regulation of the APC/β-catenin pathway has been linked to 60–80% of sporadic colorectal cancers [28].

Understanding the dynamics of Wnt signalling and other pathways requires a systems-level computational modelling approach. Only then is it likely that the behaviour of these cellular pathways, in response to specific stimuli or mutations, can be predicted [29]. A key requirement of all computational models is quantitative data on the temporal, spatial and post-translational characteristics of the critical signalling proteins and their complexes. Availability of quantitative data for modelling signalling pathways is often limited or incomplete. The sparsity of signalling data means that the modelling approach must be adapted to the availability and quality of the data [30], [31]. In 2003, Lee *et al*. [32] developed a computational model of the Wnt signalling pathway based on quantitative data from Xenopus egg extracts. Subsequent computational models for Wnt signalling [33], [34], [35], [36] have been largely based on the Lee *et al*. model; but the interpretation of predictions for mammalian systems is limited by the lack of corresponding mammalian data for the concentrations of key Wnt signalling proteins. In order to establish a quantitative basis for understanding of Wnt signalling in mammalian cells and human cancer, knowledge of the concentrations of key Wnt signalling proteins in mammalian cells is essential.

In this study we report initial estimates of the concentration of β-catenin, Axin, APC, GSK3β and E-cadherin in five mammalian cell lines (HEK293T (Human Kidney Epithelial) [37], Madin Darby canine kidney (MDCK, Normal Canine Kidney Epithelial) [38], [39], Caco-2 (human intestinal epithelial cell line from a colorectal carcinoma) [40], SW480 (Human Colorectal Adenocarcinoma) [41] and SW480APC (Human Colorectal Adenocarcinoma expressing full-length recombinant APC) [42]) in the basal (non-stimulated) state. The first four proteins are well established components of the Wnt signalling pathway (which were also measured in the Lee *et al.*'s Xenopus study [32]). E-cadherin is an important interaction partner of β-catenin in the cell-cell adhesion pathway [43], [44]. A confocal microscopy technique is developed to measure the average cell volume for each of the cell lines, allowing the calculations of the concentrations of the key Wnt signalling proteins within cells.

The results presented here demonstrate significant differences in the concentrations of Wnt signalling proteins between the Xenopus egg extracts [32] and mammalian cell lysates. A key finding in the Lee *et al*. [32] Xenopus study was the relatively low Axin concentration, compared to other measured proteins. In the Xenopus egg extracts, low concentrations of Axin create a rate-limiting step in β-catenin degradation. Here we report that, in the mammalian cell lines, the concentrations of Axin are considerably higher and are comparable to other protein concentrations. Differences in the relative Axin and APC concentrations are observed between mammalian intestinal epithelial and kidney epithelial cells. In particular, higher concentrations of Axin and lower APC concentrations are found in the kidney epithelial mammalian cells, while in intestinal epithelial cells, relatively high APC concentrations and low Axin concentrations are observed. Mammalian cells have higher β-catenin concentrations, than that observed in the Lee *et al.*'s Xenopus extract, despite having a higher Axin concentration. These mammalian experimental results provide a basis for formulating computational models of the *mammalian* Wnt pathway. The differences observed between the two systems support the need for caution when translating the Xenopus Wnt signalling model [32] into a mammalian system. Each mammalian cell systems illustrates a different aspect of normal and neoplastic Wnt signalling and should facilitate the development of progressively integrated, quantitative models of the responses of particular mammalian cells to Wnt stimulation.

## Results and Discussion

### Average viable cell compartment volumes of mammalian cells

**Figure 1:**
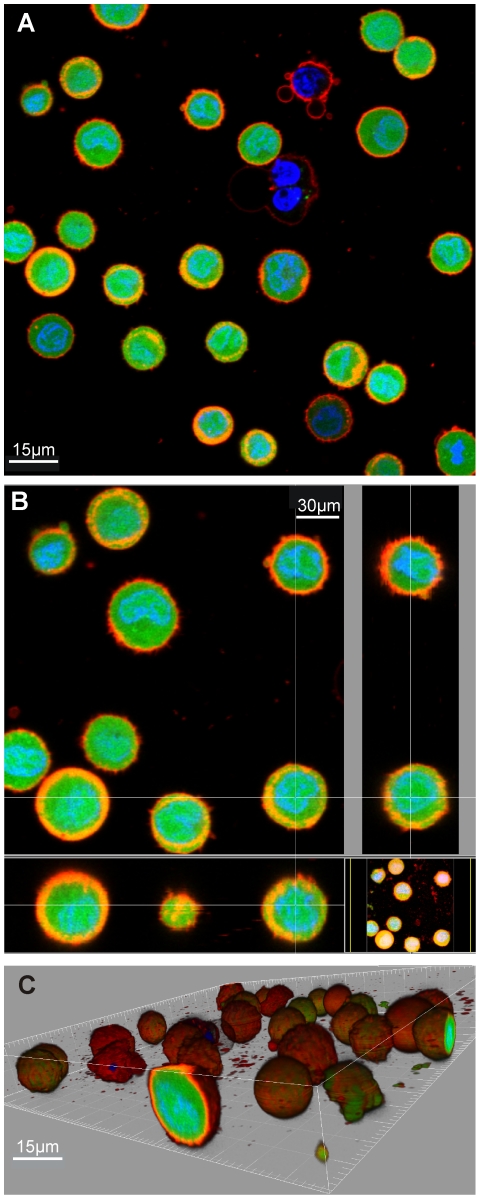
3D confocal imaging sample results (SW480APC). Trypsinised SW480APC cells stained with Calcein AM fluorescent dye (green), Hoechst 33342 nucleic acid stain (blue) and Vybrant DiI cell labelling solution (red), fluorescent marking the cytoplasm, nuclei and membrane respectively. Different overlayed fluorescent signal views of a sample of the acquired image stack are as show, namely (A) 2D sectional view; (B) orthogonal views of selected cells; (C) 3D volumetric view with blending effect.

**Figure 2:**
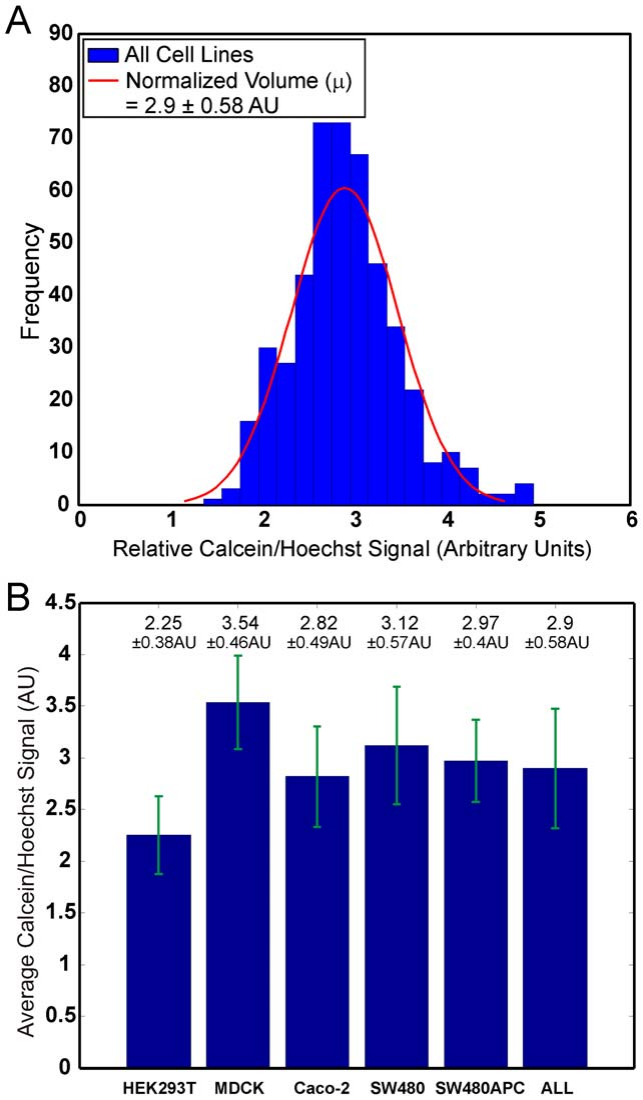
Population statistics of all cell types. Population statistics of all cells showing the (A) histograms of normalised volume (Calcein AM/Hoechst) and the (B) average normalised volume column chart. (AU: Arbitrary Units).

**Figure 3:**
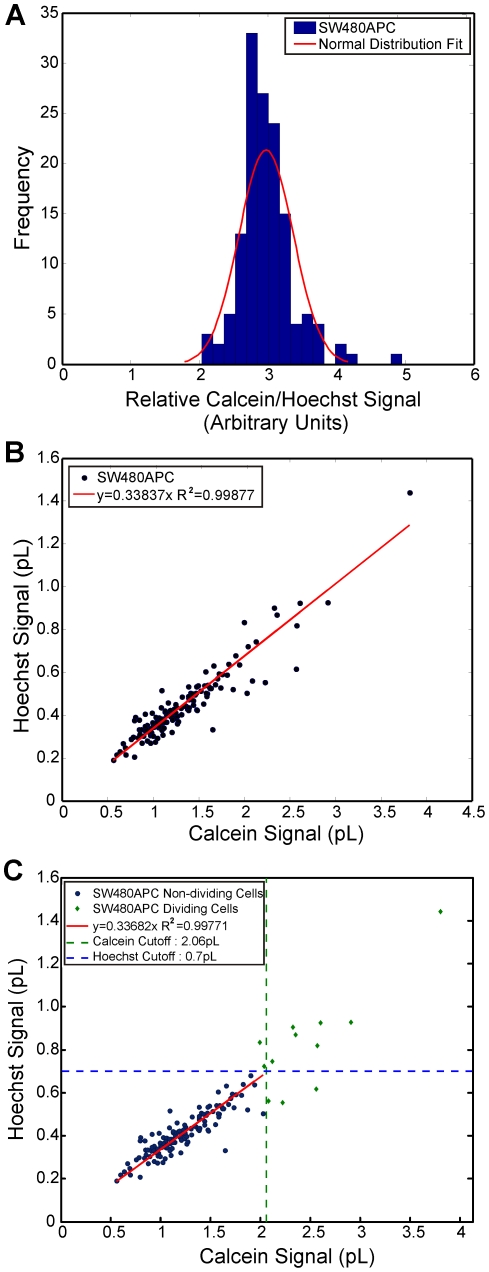
Cell population sizing and selection. Population statistics of SW480APC, showing the (A) histograms of normalised volume (Calcein AM/Hoechst); (B) Scatter plot of fluorescent marked volumes (Calcein AM vs. Hoechst) showing a good linear relationship; (C) Scatter plot of fluorescent marked volumes (Calcein AM vs. Hoechst) showing population isolation and the cut-off volumes applied. Statistical results for other cell lines are as shown in the Figures S12, S13, S14, and S15.

A protocol based on cytochemistry was developed using engineering computational tools to measure and analyse the whole cell and compartment (cytosol and nucleus) volumes of mammalian cells in the resting (or ‘steady state’) and the dividing state. The whole cell and cell compartment volumes of five mammalian cell lines were determined: at least 50 cells were analysed for each cell line. Live cells stained with fluorescent compartment markers, namely Hoechst 33342 (nuclei), Calcein AM (cytosol) and Vybrant DiI (membrane) were prepared and imaged using confocal microscopy. The acquired image stacks show that the trypsinised cells are generally spheroidal or ellipsoidal in appearance (Figure 1). After image processing and analysis, the measured fluorescent marked volumes of the cells are processed and analysed. Data analyses indicate a linear correlation between the viable whole cell volumes with the nuclei volume. First, the normalised Calcein AM and Hoechst volume ratio follows a normal distribution with a mean ratio for all the cell types of 2.90±0.58 (Figure 2, mean ratio range of between 2.2 to 3.6 for independent cell lines) implying that the cell nucleus occupies about one third of the cell volume (Figure 2A). Second, an empirical data fit between the Calcein AM and Hoechst 33342 compartment volumes produced very good R^2^ linear fits (0.78 to 0.999) (Figure 3B and Figures S12, S13, S14, and S15). These results indicate a consistent relationship between viable cell volume and the phase of cell cycle. The cell cycle phase determines the DNA content and subsequently the nuclear volume (i.e. a dividing cell also has a larger average cellular volume and thus maintains its cell size to nuclear size ratio) [45], [46], [47]. When a cell is in the S phase, DNA replication doubles the DNA content, while in M phase, the cell volume doubles [46]. These results are consistent with reports suggesting that the cell regulates and maintains an average cell size and nuclear-cytoplasmic ratio over successive generations [45], [47].

Using this relationship, the resting (non-dividing, ND) and dividing (D) populations of cells can be identified (using the selection criteria described in Text S2). Results are tabulated in Table 1. The results indicate that out of the 469 cells analysed, about 90% of the cells are not in S or M phase. The cell lines in this study have fewer than 10% of dividing cells, with the exception of MDCK cells which have a dividing cell population of about 14%. The non-dividing cell population is used for all estimates of protein concentrations. It is noted that the resultant Hoechst/Calcein R^2^ linear correlation for the non-dividing population is still reasonably correlated (see Table 1, Figure 3C and Figures S12, S13, S14, and S15, R^2^ 0.58 to 0.99).

**Table 1:** Statistics for cell population identification and isolation.

Cell Lines	*Total*	Non-Dividing Population (*ND*)	Dividing Population (*D*)	% *ND*	% *D*	*Total* R^2^	*ND* R^2^
**HEK293T**	83	76	7	91.6	8.4	0.78	0.58
**MDCK**	56	48	8	85.7	14.3	0.80	0.58
**Caco2**	119	110	9	92.4	7.6	0.99	0.71
**SW480**	72	68	4	94.4	5.6	0.80	0.68
**SW480APC**	139	127	12	91.4	8.6	0.99	0.99
**All Cell Types**	469	429	40	91.5	8.5	-	-

The volumes (calculated and marked by compartment markers) of the two populations (N and ND) are tabulated in Table 2. These results show a doubling of cell volumes for dividing cells versus resting cells in all the cell lines, as seen for each fluorescent marked volume with mean D/ND volume ratios (all cell lines) of 2.0, 1.94 and 2.0 for the three compartment markers respectively. This is consistent with the expected doubling of cell size during mitotic division. Interestingly, the D/ND volume ratio for MDCK appears to be consistently lower than the other cell lines at 1.7 while the D/ND volume ratio for SW480 appears to be higher for the Calcein AM and Vybrant DiI marked volumes compared with the respective averages. These volume measurement results indicate that Caco-2 cells have by far the largest volumes (Table 2). Among these cell lines, MDCK was the only cell line having a previous independent measurement of its cell volume. Roy and Sauvé in 1987 reported the volume of MDCK cells to be 1.8±0.2 pL [48]. This measured volume is in good agreement with the corresponding value measured in this study (1.65±0.51 pL). Furthermore, this measurement protocol enables the different populations of dividing (2.53±0.37 pL) and non-dividing (1.50±0.36 pL) cells to be isolated and measured.

**Table 2:** Summary for fluorescence marked volumes for all five cell types.

Cell Type	Marker	Total (pL)			Non-Dividing (pL) [*ND*, Resting]			Dividing (pL) [*D*]			*D÷ND* *
**HEK293T**	**Calcein AM**	1.55	±	0.51	1.44	±	0.35	2.75	±	0.39	1.9
**MDCK**	**Calcein AM**	1.39	±	0.42	1.27	±	0.29	2.15	±	0.24	1.7
**Caco2**	**Calcein AM**	2.23	±	0.88	2.08	±	0.68	4.16	±	0.83	2.0
**SW480**	**Calcein AM**	1.64	±	0.73	1.52	±	0.51	3.62	±	1.06	2.4
**SW480APC**	**Calcein AM**	1.33	±	0.49	1.22	±	0.32	2.47	±	0.50	2.0
**HEK293T**	**Hoechst 33342**	0.69	±	0.21	0.65	±	0.14	1.13	±	0.28	1.7
**MDCK**	**Hoechst 33342**	0.40	±	0.11	0.36	±	0.08	0.61	±	0.07	1.7
**Caco2**	**Hoechst 33342**	0.80	±	0.33	0.74	±	0.22	1.61	±	0.39	2.2
**SW480**	**Hoechst 33342**	0.53	±	0.2	0.50	±	0.15	1.03	±	0.27	2.1
**SW480APC**	**Hoechst 33342**	0.45	±	0.17	0.41	±	0.10	0.83	±	0.24	2.0
**HEK293T**	**Vybrant DiI**	1.97	±	0.73	1.82	±	0.51	3.66	±	0.62	2.0
**MDCK**	**Vybrant DiI**	1.65	±	0.51	1.50	±	0.36	2.53	±	0.37	1.7
**Caco2**	**Vybrant DiI**	2.81	±	1.11	2.61	±	0.85	5.28	±	1.02	2.0
**SW480**	**Vybrant DiI**	2.11	±	0.9	1.97	±	0.65	4.46	±	1.40	2.3
**SW480APC**	**Vybrant DiI**	1.70	±	0.68	1.56	±	0.45	3.18	±	0.89	2.0

*Mean D÷ND ratios of all cell types for Calcein AM, Hoechst 33342 and Vybrant DiI are 2.0, 1.94 and 2.0 respectively.

**Figure 4:**
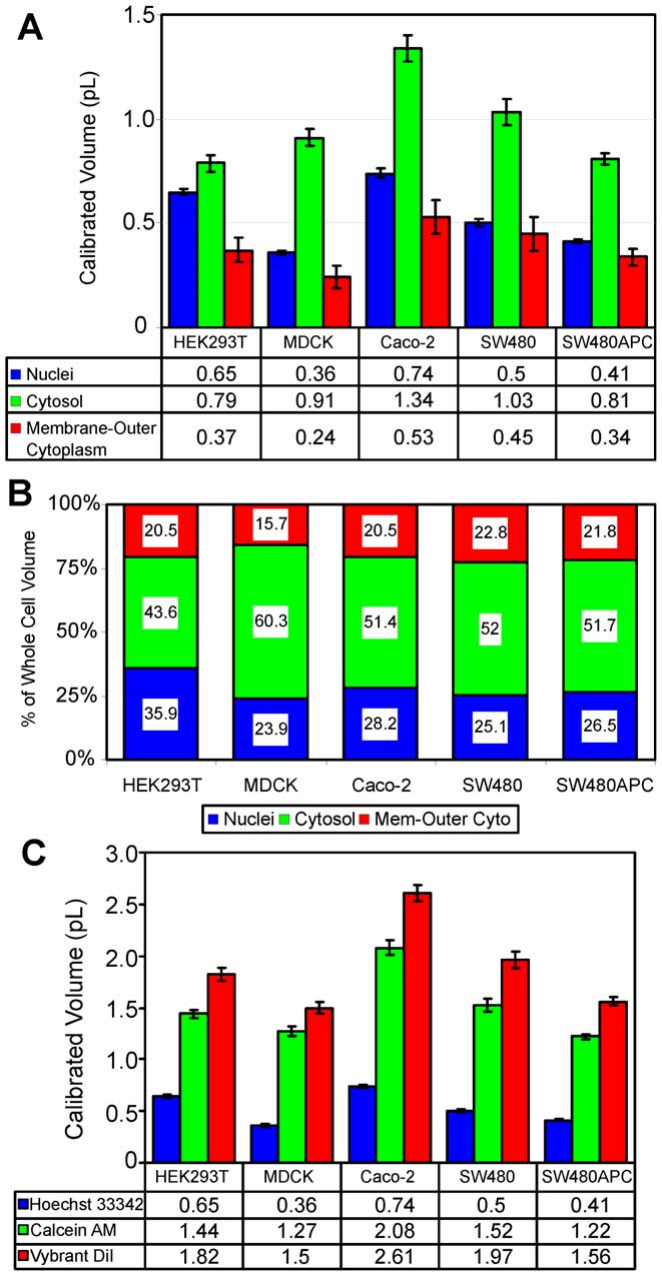
Cell volume measurement statistics for non-dividing cells. Fluorescent marked and calculated compartment volumes and distribution results showing (A) calibrated cell compartment volumes in pL, (B) distribution of compartment volume (% of total) and (C) fluorescent marked volumes in pL for non-dividing cells.

The measured compartmental volumes for non-dividing cells are tabulated in Table 3 and Figure 4A for each cell type. Note the lipid bilayer of the membrane is much smaller than the resolution of the confocal and the Vybrant DiI membrane stain localisation is not confined to the membrane. Consequently, instead Vybrant DiI membrane stain localisation is better described as labelling the membrane and near membrane cytoplasm (or outer cytoplasm). Hence we report three compartment volumes, the nuclei, the cytoplasm and the membrane-outer cytoplasm compartment.

**Table 3:** Compartmental volumes and ratios.

	Compartment Volume (pL)			Compartment Ratios
Cell line	Nuclei	Cytosol	Membrane-Outer Cytoplasm	Nuclei∶Cyto∶Membrane-Outer Cytoplasm
**HEK293T**	0.65	0.79	0.37	1.0∶1.2∶0.6
**MDCK**	0.36	0.91	0.24	1.0∶2.5∶0.7
**Caco-2**	0.74	1.34	0.53	1.0∶1.8∶0.7
**SW480**	0.50	1.03	0.45	1.0∶2.0∶0.9
**SW480APC**	0.41	0.81	0.34	1.0∶2.0∶0.8

Approximately half of the cellular volume of non-dividing cells is associated with the cytoplasm (Figure 4B), while nuclei and membrane-outer cytoplasm compartments contribute equally to the remaining volume, i.e. the Nuclei∶Cytosol∶Membrane-Outer Cytoplasm (N∶C∶M) ratios of the different cell lines were approximately 1∶2∶1 (Table 3); however, the MDCK and HEK293T's compartment distributions were 1∶3∶1 *i.e.* a larger cytosolic compartment, and *1∶1∶0.5 i.e*. a larger nuclear compartment, respectively.

The confocal quantification technique not only provided consistent whole cell volume quantification, it also provides the cell compartment volume data. Cell compartment data are crucial for modelling of the intracellular pathways as protein concentrations are rarely evenly distributed throughout cells. Other advantages of this technique include the ease of application with relatively simple staining, imaging and cell selection steps involved. Furthermore, the analytical quantification has been partially automated in MATLAB. This procedure can be further developed for extensive and bulk quantification of large datasets. The availability of such tools to biochemist and/or computational biologists will provide a fast and reproducible alternative to acquiring crucial quantitative data needed for systems and computational modelling. In addition to the absence of whole cell volume data for the mammalian cells, there is also an absence of compartment volume data in the current literature. In recent years, it has become clearer that many of the mechanisms in the cellular pathway involve translocation between compartments within the cell. To understand and model the relative changes and translocation of proteins between compartments, an understanding of the levels of specific proteins in different regions or compartment of the cell is needed. These proteins concentrations are crucial for estimating the reaction rates in the computational models of signal transduction pathways.

### Concentrations of Wnt signalling proteins in five mammalian cell lines

**Figure 5:**
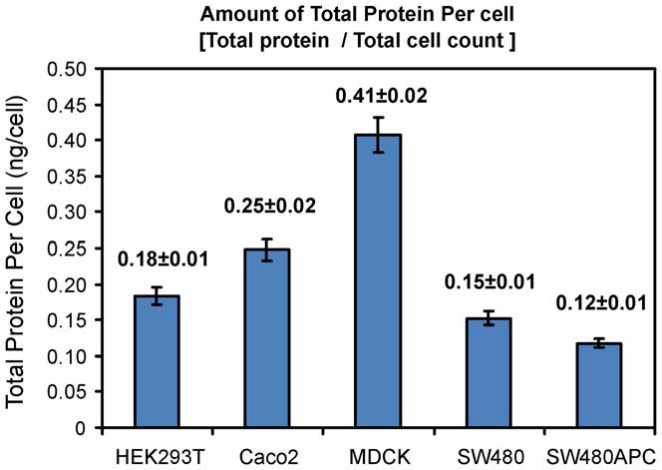
Total protein per cell for each cell line. Error bar: Root Mean Squared Error (SEM).

The total protein amounts per cell, as shown in Figure 5, are calculated from the quantitative immuno blots and cell count experiments. Typical western blots for the five proteins investigated in this study are as shown in Figure S5. It is un-surprising that some of the proteins exhibit closely located multiple bands on the blots due to probable degradation products. These multiple bands appear to be more pronounced in β-catenin and E-cadherin blots. In this study, the higher or more pronounced bands at the molecular weights for the full-length functional proteins are used for our measurements. In the case of APC in the intestinal epithelial cell lines, the truncated form of APC (ΔAPC) [49] is quantified and it should be noted that for SW480APC, the level of full length (wild-type) APC [42] is too low for quantitation. In kidney epithelial cells, full length (wild-type) APC can be detected and quantified. Axin expression is viewed as a doublet band for all the cell lines.

**Figure 6:**
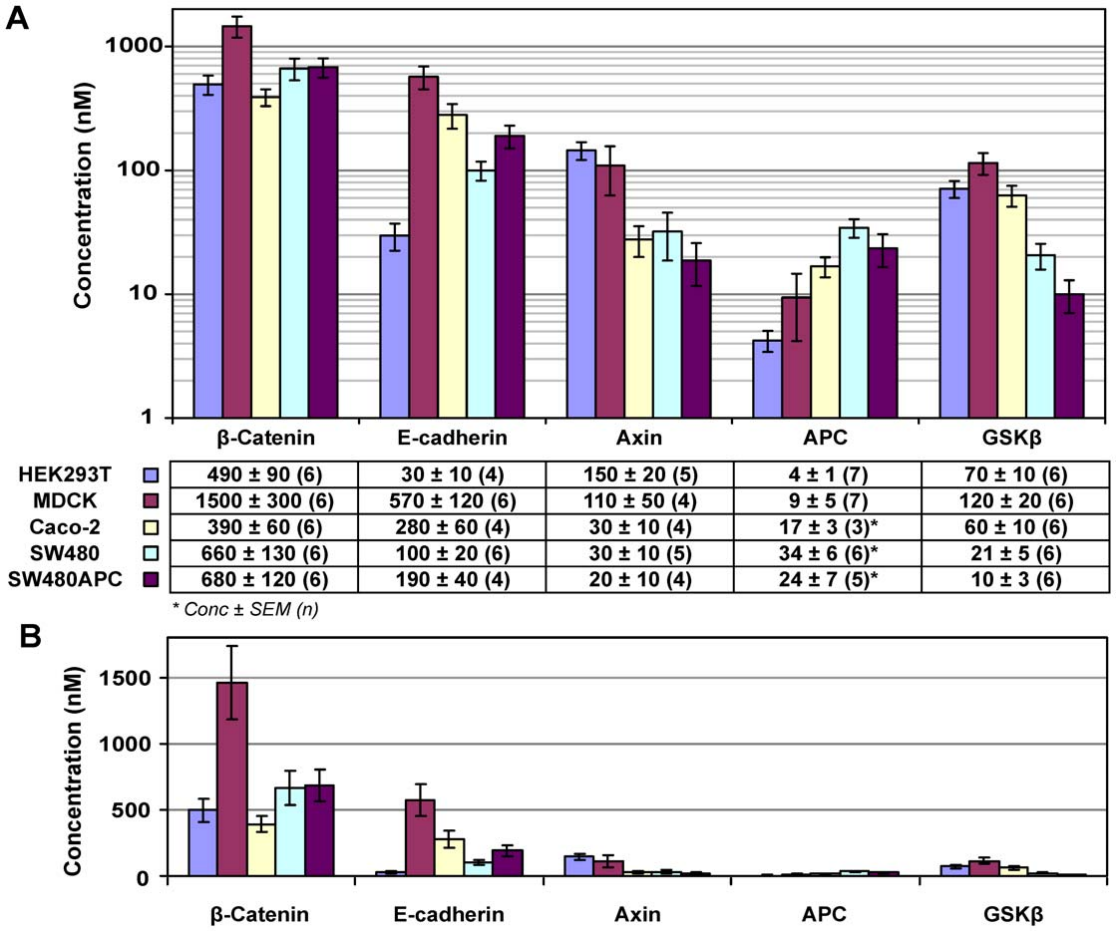
Key protein concentration distribution in mammalian cells. (A) Average concentration of key Wnt proteins and E-cadherin of the five mammalian cell lines. (B) Figure redrawn in linear scale, reiterating the significant difference in concentrations between the proteins measured and the effect of log scales on perception of concentration differences. *Note that the APC measured in Caco-2, SW480 and SW480 are ΔAPC. The amount of wild type APC is too low to be detected and the measurement here is for ΔAPC. In SW480APC, approximately 95% of the total APC is ΔAPC (unpublished data) (Data: Concentration in nM ± SEM (n repeats), Error bars: SEM).

Quantification results show that MDCK cells have the highest amounts of total protein per cell among the cell types investigated. Subsequently, the concentrations of the four key Wnt proteins and E-cadherin were calculated and are summarised in Figure 6 with molecules per cell calculations tabulated in Table 4. From the concentration data, two groups of proteins can be distinguished. For all cell lines, β-catenin and E-cadherin concentrations are significantly higher than the other three proteins (APC, Axin and GSK3β), with the only exception being the E-cadherin concentration of HEK293T cells. In HEK293T, there is a low level of E-cadherin compared to the other cell lines. This low level of E-cadherin in the HEK293T cells might be due to the presence of N-cadherin [50], [51] (data not shown as not measured in this study). On the other hand, HEK293T cells have the highest Axin concentration among the cell lines. The MDCK cells have the highest E-cadherin concentration (570 nM), β-catenin (1500 nM) and GSK3β (120 nM) concentrations among the various cell lines. These concentrations are significantly higher than the corresponding concentrations in the human cell lines.

**Table 4:** Molecules per cell count (×1000).

	HEK293T	Caco-2	MDCK	SW480	SW480APC
β-Catenin	540	610	1400	780	640
E-cadherin	30	440	510	100	180
Axin	160	50	99	40	20
APC	4	27	8	40	23
GSK3β	80	90	110	25	10

**Figure 7:**
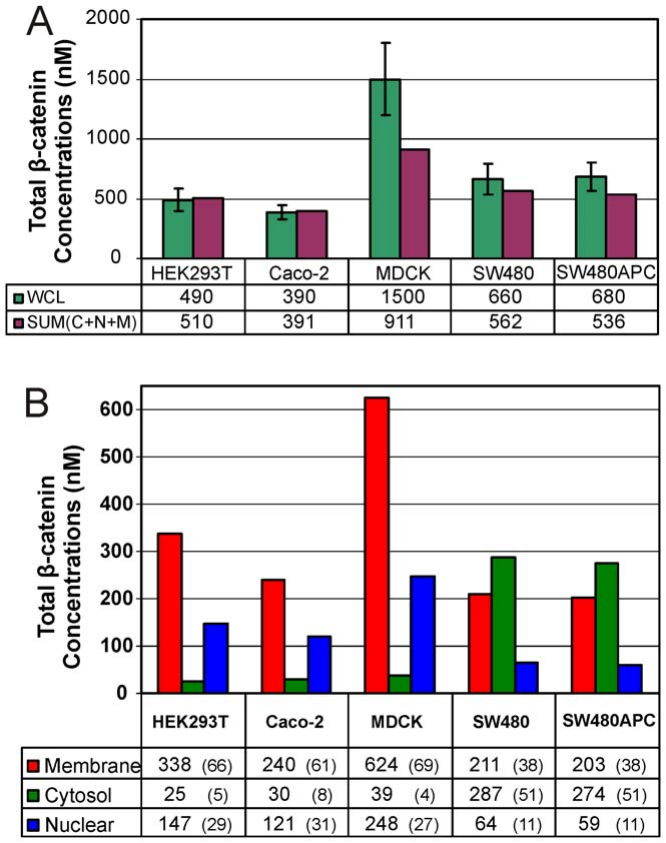
Sub-cellular distribution of β-catenin in mammalian cells. (A) Compartment concentrations for β-catenin in HEK293T, Caco-2, MDCK, SW480 and SW480APC cells. Note: Percentage of whole cell β-catenin concentrations is in parentheses. (B) Protein recoveries (Sum of compartment proteins) from the fractionation experiment compared with the measured whole cell lysate.

Measurements of total β-catenin concentrations in the functional pools or compartments (nuclear, membrane and cytosol) of the cell lines are as shown in Figure 7. Summations of the compartment protein concentrations correlates well with the whole cell lysate concentration measured, with the exception being MDCK where loss (Figure 7A) is observed due to the fractionation process. Figure 7B shows that for HEK293T, MDCK and Caco-2, the majority of the β-catenin (>65% of total) is in the membrane with about 4–8% in the cytosol and the remainder (27–31%) in the nucleus. The high membrane association of β-catenin is highly likely to be in complex with the cadherin consistent with the adhesive role of epithelial cells. Interestingly for SW480 and SW480APC, most of the β-catenin is in the cytosol (51%) with 38% in the membrane and a lower nuclear β-catenin (11%) which is significantly different from the other three cell lines.

It should be noted that for SW480 and SW480APC cells the culture conditions (i.e. confluency levels) have a significant impact on the consequent spatial localisation of β-catenin. This effect has been investigated briefly using both sub-cellular fractionation approach and a confocal imaging quantitation (see Figure S10 and Text S3). This dependency of β-catenin compartment distribution on cell confluency can be observed in immunofluorescent images and 3D quantification analysis (compartmentalising the nuclear and non-nuclear signal) of these cells taken at different levels of confluence. From the image analysis, when the cells are less confluent, the majority of the β-catenin is in the nucleus compartment (Figure S10E). In the above fractionation experiment, the β-catenin compartment concentrations are obtained from confluent cultures (>90%), in-line with the conditions of the whole cell lysate preparations. The compartment distributions for less confluent cells have also been investigated (see Figure S10F–H); there is a higher percentage of nuclear β-catenin and lower percentage of membrane associated β-catenin. This observation is in agreement with the imaging analysis results. This confluency dependency is an important consideration when investigating protein dynamics in SW480 and SW480APC cells.

Further to that, “active” β-catenin (ABC) concentrations [52], [53] and distributions in the nuclear, membrane and cytosol of “active” β-catenin for theses cell lines was also investigated (Figure S11). There is variation of total and sub-cellular distributions of “active” β-catenin between the different cell lines. The “active” β-catenin is higher in SW480 and SW480APC cell lines. The culture conditions affect the sub-cellular distribution of “active” β-catenin: there is increasing nuclear and decreasing cytosolic levels with decreasing cell confluency. The level of “active” β-catenin is higher in SW480 than SW480APC cells at low cell density.

**Figure 8:**
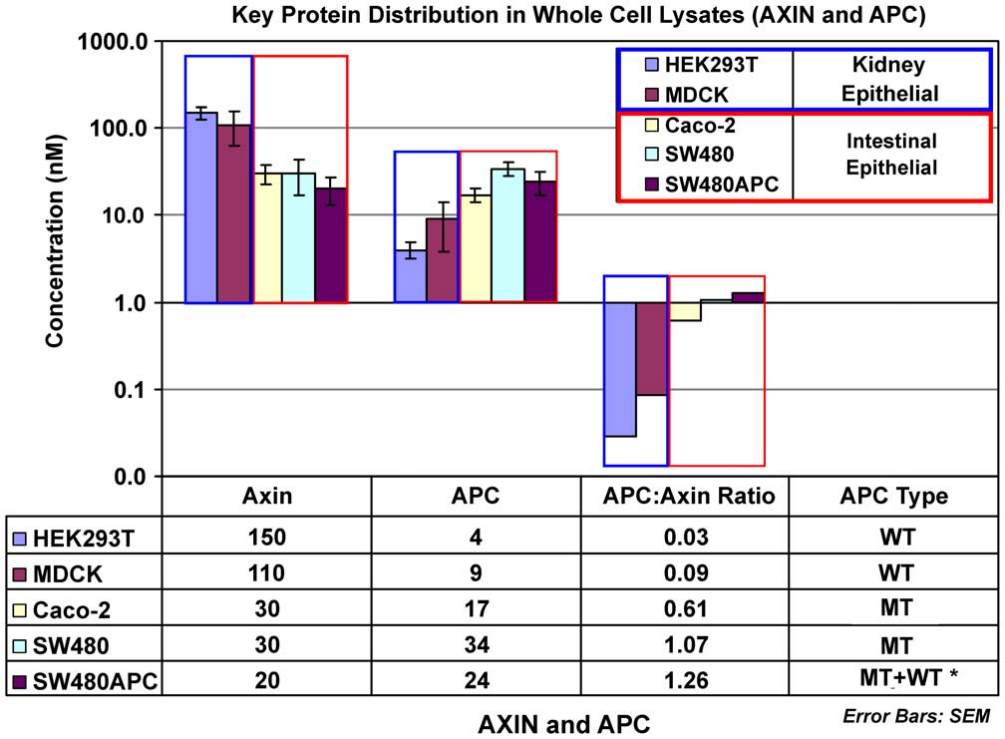
Concentrations of Axin and APC in mammalian whole cell lysate. The data is displayed in log scale. * Note that for SW480APC, as the level of wild-type (WT) APC is too low to be detected in this set of experiments, the measurements made in this study comprised that of mutant (MT) APC only. Error Bars: SEM.

Analysis of intestinal epithelial cells (Caco-2, SW480 and SW480APC) and kidney epithelial mammalian cell lines (HEK293T and MDCK) reveals a pattern between the key scaffold proteins Axin and APC levels, whereas no trend is seen in the β-catenin concentrations between these two cell groups. The initial observation is that Axin and APC are present at comparatively low concentrations in intestinal mammalian cells. The absence of a pattern in β-catenin levels is unexpected as the β-catenin levels are hypothesized to be modulated by the degradation complex (which includes APC and Axin). As such, higher wild-type Axin or APC levels should imply a corresponding lower β-catenin levels and vice versa, but this is not observed here. It is also noted that the concentration of Axin is higher than that of APC in the kidney cells; however, in the intestinal cells APC concentrations are higher than Axin concentrations. The significantly higher level of Axin than APC, for kidney mammalian cell lines, as compared to intestinal mammalian cell lines, is shown by the small APC∶Axin ratio (<0.1) for kidney mammalian cell lines in contrast to an APC∶Axin ratio greater than 0.5 for intestinal mammalian cell lines (see Figure 8). It is noted that SW480APC has the highest APC∶Axin ratio (1.26) among the cell lines. It should be highlighted that the Caco-2 and SW480 cell lines have mutant APC gene leading to the expression of truncated forms of APC (ΔAPC), with reduced ability to form signalling complexes.

Caco-2 has a single APC with a nonsense mutation at codon 1367, a C to T transition changing Gln (CAG) to a stop codon (TAG) [54], [55], [56], [57]. SW480 gene has only one APC allele truncated at codon 1338 [49], [56], [58]. Further, SW480 cells express only ΔAPC [49] while SW480APC are SW480 cells with stable ectopic expression of wild-type APC [42]. However, the amount of wild type APC was found to be too low to detect and the SW480APC measurement reported here is for ΔAPC (which represents about 95% of total APC in the SW480APC cells (Dr Maree Faux, unpublished data)). The truncated APC of SW480, SW480APC and Caco-2 retains all the 15 amino-acid (aa) repeats but only one 20 aa repeat [55]. This loss of C-terminal sequences leads to a loss of binding functions for nuclear import, microtubule binding, EB1/RP1 binding, and Axin binding sites. Depending on the degree of truncation, the truncated APC (ΔAPC) may lose most of, if not all of, its β-catenin binding [59]. All these processes could cause changes to the regulation and functions of the truncated APC protein and potentially downstream proteins that it regulates. One example of such a potential regulation could be an increase in E-cadherin expression with the stable expression of full-length wild type APC in SW480APC cells, as compared to SW480 (which expresses ΔAPC), which suggested a role for APC in the regulation of E-cadherin localization [42]. The increase in expression of E-cadherin is observed in this study (that is, the concentration of E-cadherin in SW480APC cells is 190 nM as compared with 100 nM for SW480 cells, Figure 6).

These observed differences in Axin and APC concentrations between kidney and intestinal mammalian cells has not been reported previously and will be critical information in the development of mammalian Wnt pathway models.

### Differences in Key Wnt Signalling Protein Concentrations between Mammalian Cells and Xenopus Egg Extracts

**Figure 9:**
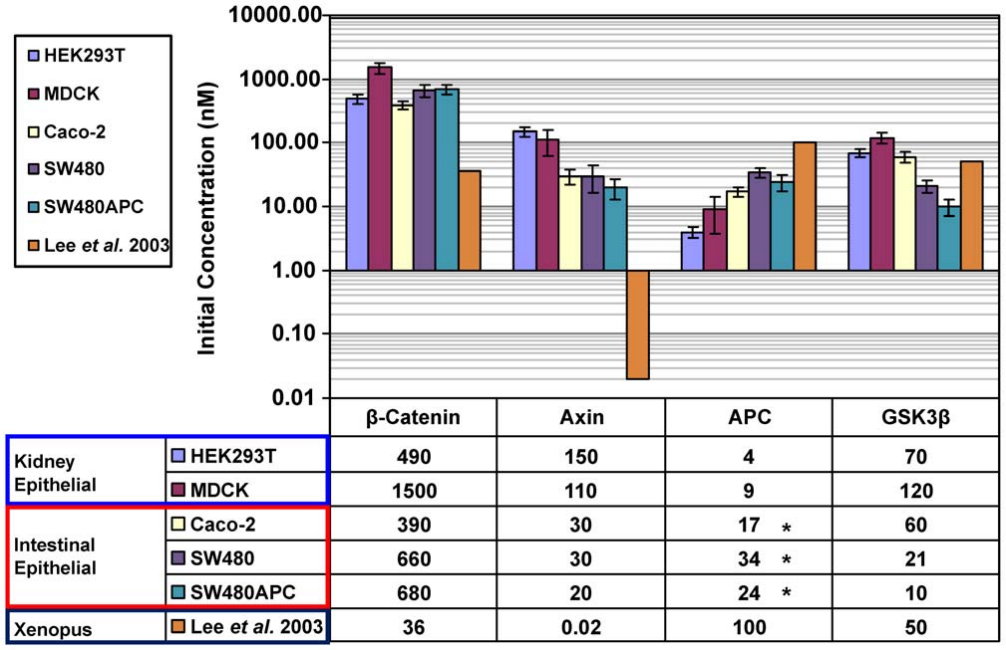
Key protein concentrations in different cell lines. Comparison of key protein concentrations (β-catenin, Axin, APC and GSK3β) between the Xenopus extract (Lee *et al*., 2003), two kidney epithelial mammalian cells (HEK293T and MDCK) and three intestinal epithelial mammalian cells (Caco-2, SW480 and SW480APC). * ΔAPC measured in Caco-2, SW480 and SW480. For SW480APC, as the level of wild-type APC is too low to be detected in this set of experiment, the measurements made in this study comprised that of ΔAPC only. The data is displayed in log scale.

The total protein concentrations for β-catenin, Axin, APC and GSK3β for the Xenopus egg extract and the WCL from the kidney epithelial mammalian cell lines, (HEK293T and MDCK) and three intestinal epithelial mammalian cells lines (Caco-2, SW480 and SW480APC) are shown in Figure 9. Lee and co-workers [32] reported the total concentration of APC, GSK3β, β-catenin and Axin for the Xenopus egg extract [32]. Their estimations of protein concentrations were obtained using similar techniques as to those presented here, i.e. quantitative Western blot analysis. In Lee *et al*. (2003), the levels of APC and GSK3β were assumed to be maintained at a comparatively high level as biochemical degradation experiments indicated reasonably slow turnover of APC and GSK3β with no clear degradation observed within 3 hours. Therefore no synthesis or degradation for the two proteins is explicitly parameterized in their model.

In view of establishing a better understanding of the differences in protein levels between the two cellular systems, comparison were made between Lee *et al.*'s estimations and the results acquired in this study. Several differences were noted. First, comparing kidney epithelial mammalian cells with Xenopus, the β-catenin concentrations are 10 to 40 times *higher* in the kidney epithelial mammalian cells than that reported in the Xenopus egg extracts. Levels of GSK3β are similar in both systems. There is a marked difference in the concentrations of Axin. Lee and colleague (2003) proposed that Axin is the rate limiting factor in the Xenopus Wnt signalling pathway, as the measured total concentration of 0.02 nM are two to three orders magnitude lower than the other measured protein concentrations in the Wnt signalling pathway. Certainly Lee *et al*.'s Axin concentrations were much lower than the 110–150 nM range detected in mammalian cells, and yet we see that β-catenin concentrations are higher in mammalian cells despite Axin presumably no longer being rate-limiting. Furthermore, no substantial differences in β-catenin between kidney epithelial mammalian and intestinal epithelial mammalian cells are observed in spite of the differing levels of Axin. These observations are therefore inconsistent with the dogma that β-catenin degradation, facilitated by the degradation complex, is limited by the level of Axin. The situation is further complicated by an unexpectedly low APC level in kidney epithelial mammalian cells. One possible explanation for this discrepancy between the two cellular systems may be due to the presence of self-associated aggregations or vesicles of Axin in the cytoplasm in HEK293T and MDCK cells known as puncta [19], [60], [61] as indicated in recent studies. Puncta formation may modulate the overall concentrations of soluble Axin (in the cytoplasm) possibility by means of aggregation or reduction in degradation by shielding. Another possible explanation is the observation that the total concentration of APC in the kidney epithelial mammalian cell lines was more than 10-fold lower than Lee's values (where they postulated that APC was in abundance and unlikely to change significantly with time). That is, a comparatively lower APC may offset the higher Axin in affecting mammalian β-catenin concentrations.

Comparing intestinal epithelial mammalian cells with Xenopus, again the β-catenin concentrations are *higher* (10 to 20 times) in the intestinal epithelial mammalian cells than the Xenopus egg extracts. Similarly to kidney epithelial mammalian cells, Axin levels are even more elevated (1000–1600 times higher) in intestinal epithelial mammalian cells than in the Xenopus extracts. In contrast, APC levels are 3 to 6 times lower in intestinal epithelial mammalian cells. It is surprising that the difference in β-catenin levels between kidney epithelial mammalian and intestinal epithelial mammalian cells are not as substantial as one would have expected. This implies that the pool of β-catenin regulated by the Wnt pathway might be small in comparison to the total amount of β-catenin protein. The GSK3β in Caco-2 cells are similar to the Xenopus extract, whereas in SW480 the GSK3β levels are 2 to 5 times lower than the Xenopus extracts.

Overall, APC concentrations in these cell lines are significantly lower than that reported in the Xenopus egg extract by Lee *et al.* (2003). This is surprising considering APC is widely considered to be a tumour suppressor protein and therefore is expected to be at a significant level in order to serve that particular function. Again despite differing levels of APC between kidney epithelial mammalian cells and intestinal epithelial mammalian cells, there are not substantial differences in β-catenin levels, adding to the complication of the roles of Axin and APC in β-catenin modulation or the subset of β-catenin affected by this modulation.

In general, mammalian cells (kidney epithelial and intestinal epithelial) have lower APC and higher Axin and β-catenin levels than the Xenopus extract. One might expect these differences in these key protein concentration to substantially change the dynamics of signalling through the Wnt pathway. All of these observed differences might be due to the different cellular systems involved (mammalian vs. amphibian) or the different functional needs (epithelial vs. reproductive cells) of the tissues, and so for the cells of different tissue origin. Xenopus extract was based on the cytoplasm of Xenopus egg while the mammalian cells are epithelial cells. Nonetheless, these results highlight the care needed in using data from a range of species or even cell types within a species and the requirement of more species specific quantitative data. These steady state protein quantifications will be crucial data for progressive computationally modelling of the mammalian Wnt pathway.

### Calibration Analysis of Lee *et al*. 2003 Model for mammalian cells

Lee *et al*. proposed a computational model (Figure 2A of Lee *et al.* 2003 [32]) for the interactions and dynamics of the core components of the Wnt signalling pathway in the Xenopus egg extract system. In order to determine if Lee *et al*. 2003 model [32] is applicable for mammalian cells, the model needs to be tested with the new mammalian data obtained here. Possible computational validation tests include adjustments of reaction rates, initial conditions or fluxes. In this study, the Lee *et al*.'s model is tested in its response to adjusting the initial protein concentrations to the levels found in mammalian cell extracts only.

**Figure 10:**
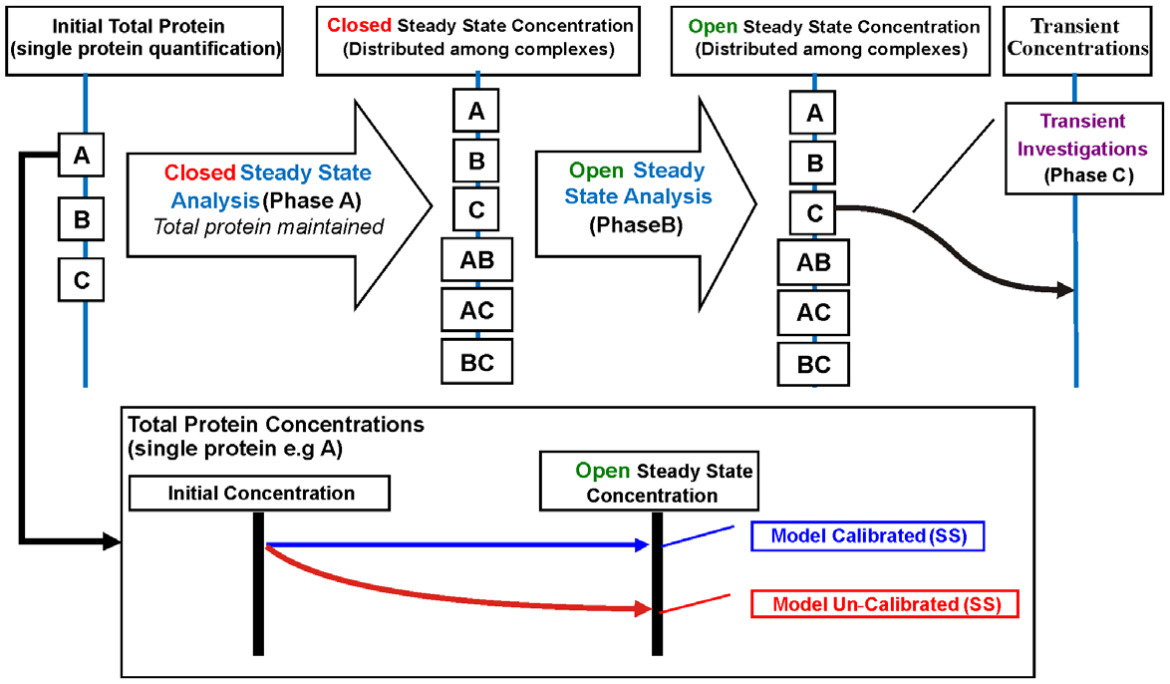
Computational Modelling Steps and Model Calibration Test Requirement. Typical computational modelling involves initial steady state analysis: Phase A, initial concentration to steady state protein redistribution in a closed system (no input and output from the system); Phase B, steady state protein redistribution in an open system and a subsequent transient analysis (Phase C, steady state to perturbation time-course). Phase A and B involves redistribution of proteins among the protein complexes while Phase C applies the redistributed concentrations to conduct transient investigations. Phase A and B are initial pre-requisites for a calibrated model whereby the total protein concentration for each protein should be maintained at steady state in an open system (inset).

The Lee *et al*. 2003 model [32] was reconstructed (in its complete form with the full set of ODE equations) and allowed to run to steady state under specific conditions. Specifically, the reaction rates from the original Lee *et al.* model were retained, including the production and degradation rates of various pathway components, such that the only change made is the use of mammalian whole cell concentrations measured here as the initial concentrations for the simulations. Steady state analysis is then conducted in two phases (A and B) based on that described by Lee *et al.* 2003 [32] with modifications (see Figure 10). In Phase A, the model is simulated without Wnt stimulation (Wnt = 0) as a closed system (i.e. no input and output fluxes) and the model allowed to run to let the respective proteins distribute among the various protein complexes. New steady states are so obtained from the initial total concentration of each protein. In Phase B, using the newly calculated steady states as initial concentrations, again the model is simulated without Wnt stimulation but this time as an open system (i.e. all reactions and external fluxes applied). The simulation allowed the respective proteins to again be redistributed among the complexes and the steady state levels for the open system calculated. For the model to be open-system, steady-state calibrated, the total initial concentration of each protein, after completing Phase B, is expected to be maintained at steady state. Only upon calibration of the open-system, steady-state (OSSS) should the analysis proceed on to subsequent temporal tests and analysis (Phase C, Figure 10).

**Figure 11:**
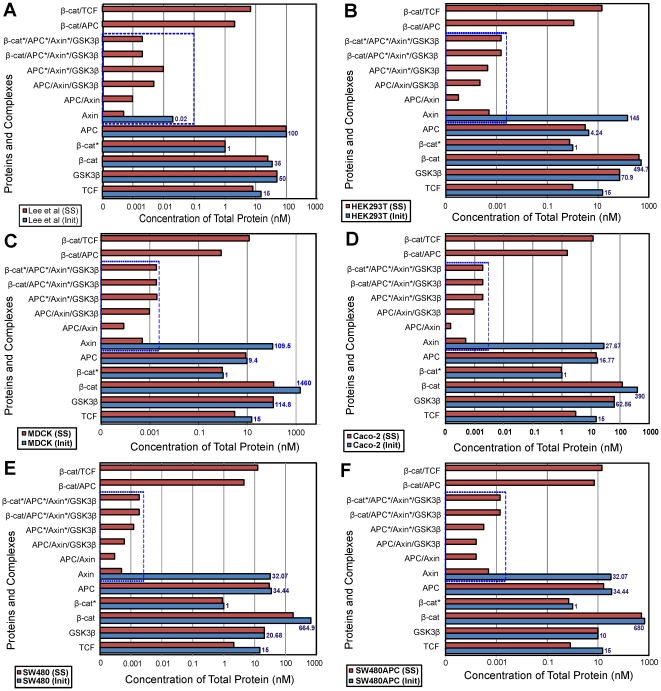
Steady State Analysis of protein complex for different cell lines. Initial to steady state protein redistribution among the interacting complexes of the Wnt signalling pathway for the different cell lines. (A) Xenopus (B) HEK293T (C) MDCK (D) Caco-2 (E) SW480 and (F) SW480APC. Note: Concentrations of protein and complexes involving Axin (in dotted box) are significantly low in all cell lines.

Comparison of open-system, steady-state results (Phase A and B, Figure 11) for Lee *et al*. model using mammalian concentrations indicate significant differences. Figure 11 presents the initial and open-system steady-state predictions of the concentrations of the different components of the pathway in each cell type and shows how the proteins redistribute among the various complexes. A clear difference in steady state response is obtained using the mammalian data compared to that for the Xenopus data. This suggests that the Lee *et al*. model is not representative of mammalian cells. At a minimum, additional calibration of rates of production or degradation, reaction rates, or new reactions would be required to obtain agreement between observed total concentrations and model simulations.

**Figure 12:**
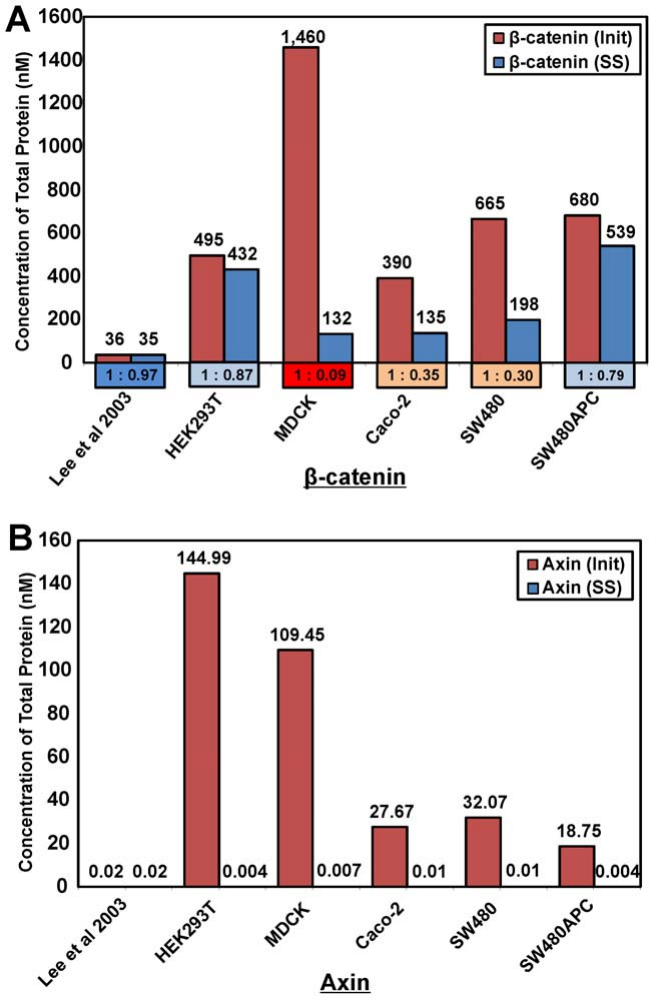
Total protein concentrations of β-catenin and Axin. Initial and Steady State total concentrations for Xenopus egg extract (Lee *et al.*, 2003) and whole cell lysate (WCL) from the mammalian cell lines. As expected, APC and GSK3β remained stable (results not shown) due to the lack of input and output fluxes in the Lee *et al.*, 2003 model. Notes: For β-catenin, the ratio of total initial to steady state (I∶SS) concentrations are given in the box below the bar for the respective cell line. Total protein concentration implies summation of concentrations of free and all protein complexes containing the specific protein.

Using the Lee *et al.* model [32], the predicted steady state concentration of *free* Axin for all cell lines is very low (∼0.5 pM). Furthermore, concentrations of proteins for the majority of the complexes in the degradation cycle are low and primarily limited by the low level of steady state free Axin. Figure 12 shows the free protein and complexes levels for each protein in the system. The level of *total* Axin in all the cell types tends toward a very low level (<0.02 nM). The steady state level of β-catenin is lower than the initial starting total concentration (as can be seen from the initial to steady state concentration (*I∶SS*) ratios. For HEK293T and SW480APC, more than 75% of the initial total β-catenin concentration is retained at steady state, while Lee *et al.'s* (2003) model retained 97% of the initial total protein. However for MDCK, Caco-2 and SW480, less than 40% of the initial protein concentration is maintained. The *I∶SS* ratio for MDCK is particularly small at 1∶0.09 with only 9% of the initial β-catenin protein retained. It is noted that the low *I∶SS* ratio appears to correspond with smaller SS Axin level (<0.004 nM) and higher GSK3β concentration (both components of the degradation complex). As no input and output fluxes are present in the Lee *et al.* (2003) model [32] it is expected that the predicted concentrations of APC and GSK3β remained stable (results not shown).

Regardless of the set of initial concentrations (Xenopus or mammalian), the total steady state concentration of Axin is predicted to be extremely low (<0.02 nM) in the Lee *et al.* (2003) model. This low Axin level means that there would be a very low and perhaps limiting concentration of degradation complex available to degrade β-catenin. This Axin limitation can be further substantiated by the low levels of protein complexes formed (<0.0005 nM) for the different cell lines (Figure 11)

To test the uniqueness of the steady state solution for particular initial concentrations, simulations were also repeated for a different pathway to the open-system steady-state, namely, from the initial concentrations directly to the open- system steady-state (Figure 10). In other words, Phase A (closed system) was not performed prior to Phase B (open system). The same final steady state solution is obtained using the one stage or two stage protein redistribution methods (Figure 10). It should be noted that Lee's model parameters [32] were calculated from steady state solutions of the derived system equations based on their initial total concentrations. As such the parameters are optimised for the Xenopus egg extract initial concentrations with constant APC and GSK3β, along with a very low Axin levels. The production and degradation terms for β-catenin and Axin were optimised for the Xenopus egg extract experiments. The differences in observed concentrations of these proteins in the mammalian cell extracts imply that the turnover rates (i.e. rates of production and degradation) for β-catenin and Axin are different in mammalian cellular systems. The low Axin concentration may well be specific for the Xenopus egg. The applicability of this condition to mammalian cells is questioned in this study and by others [62].

In the case of mammalian cells, Lee *et al*.'s model has to be re-calibrated using the mammalian experimental data as a prerequisite for further analysis. Therefore, optimisations of the reactions constants, turnover rates or reaction topology will be required. This is the subject of investigation of our subsequent work which includes experimental analysis and computational predictions of the temporal behaviours of the Wnt signalling pathway components in several mammalian cells. Further, temporal data required to validate β-catenin dynamics in the mammalian whole cells were acquired to calibrate and validate the computational models (manuscript in preparation).

Lee *et al*.'s model was the first comprehensive computational model of the Wnt pathway and is notable for its successful integration of computational-and experimental approaches. It has triggered several subsequent computational studies that extended the Lee *et al.* model [33], [34], [35], [36]. It should however be noted that most of these extensions have been based on the parameters from the Xenopus cell free system without experimental validation in the systems being studied. From our results, it is clear that the concentrations of the various components of the pathway are different for particular cell types and species. So it should be expected that the Wnt pathway display substantially different dynamics for different cell types. This has important consequences for any conclusions drawn for models of Wnt signalling in human systems based on the Xenopus parameters. In particular, the higher concentrations of Axin in mammalian cells (20–150 nM) than in the Lee *et al.* model (0.02 nM) requires careful interpretations of Axin-mediated degradation kinetics. Preferably relevant data should be acquired to enable model validation.

Two interesting studies on Wnt modelling were published in recent years by van Leeuwen *et al.* [63]. They first developed a mathematical model of the Wnt pathway that incorporates dual roles for β-catenin in adhesion and transcription functions. They introduced aspects of compartmental modelling by distinguishing different conformations of β-catenin with different affinities for E-cadherin. They subsequently applied the model to a multi-scale computational model of intestinal crypt dynamics, i.e. a mammalian system, linking the different levels of spatial organisation for cellular signalling, cell biology and tissue organisation towards a systems approach for understanding intestinal biology [63], [64]. These studies highlight the increasing emphasis of intracellular spatial considerations to understanding and modelling of complex biological pathways. Furthermore, recent reports have described the distinction of specific spatial localisation of key Wnt signalling proteins in the cell, including GSK3β sequestering in the multivesicular bodies for functional Wnt signalling [65], Axin in sub-cellular puncta [19], [66], [67], APC with microtubules and β-catenin in the cytoplasm [68] and at the membrane [19] while β-catenin has been found at the membrane, cytoplasm and nucleus. These spatial localisations of key proteins have to be carefully considered for more advanced future models of the Wnt signalling pathway. However to date measurement techniques to obtain the quantitative compartmentalization data have been lacking. The 3D-confocal microscopy measurement technique and the data described and acquired in this study therefore provided an excellent platform for spatial data acquisition and to facilitate more in-depth modelling of signalling pathways. Specifically, with the development of quantitative imaging technique to compartmentalise cells, a move towards an experimental-computational compartment model of Wnt signalling in mammalian cells is now within reach.

## Materials and Methods

### Cell Cultures and Treatments

Mammalian cell lines were used in this study: HEK293T, a human kidney epithelial [69], Madin Darby Canine Kidney (MDCK) a normal canine kidney epithelial (sub-clone obtained from Anne Ridley, Ludwig Institute for Cancer Research, London, UK. [70]), Caco-2 (C2BBe1 clone, #CRL-2102, American Type Culture Collection, Rockville, MD) a human intestinal epithelial cell line derived from a colorectal carcinoma, SW480 a human colorectal adenocarcinoma [71] and SW480APC (a human colorectal adenocarcinoma expressing full length APC) [42]. HEK293T and MDCK were grown in Dulbecco's modified Eagle's medium (DMEM) supplemented with 10% fetal calf serum (FCS). SW480, SW480APC and Caco-2 were grown in GIBCO's RPMI media supplemented with 10% FCS, Thioglycerol (1.075 µg/ml), Insulin (1.14 µg/ml) and Hydrocortisone (1 µg/ml)). All cells investigated in this study are non-stimulated (basal state).

### Antibodies, Fluorescents Cellular Markers and Reagents

The following primary monoclonal antibodies were used in this study: anti-GSK3β (Transduction Laboratories, mouse G22320, BD Biosciences, San Jose, CA), anti-β-catenin (Transduction Laboratories, mouse 610153, BD Biosciences, San Jose, CA), anti-Active-β-catenin (Millipore, mouse clone 8E7, cat#05-665, Temacula, CA), anti-E-cadherin (Transduction Laboratories, mouse 610181, BD Biosciences, San Jose, CA), anti-β-tubulin (mouse cat#560381, BD Pharmingen™, BD Biosciences, USA). The following primary polyclonal antibodies were used: anti-APC (H-290, Santa Cruz Biotechnology, Santa Cruz, CA) and anti-Axin (rabbit cat#34-5900, Zymed Laboratories, CA) antibodies. The following secondary antibodies were used: goat anti-mouse IRDye 800CW (926–32210, LICOR Bioscience, Lincoln, NE) and anti-rabbit IRDye 800CW (926–32211, LI-COR Bioscience, Lincoln, NE).

The following cell labelling solutions were used in this study: BD™ Calcein AM Fluorescent Dye (cat# 354217 BD Biosciences, San Jose, CA) used as a green fluorescent live cell marker [72]. Calcein AM is a non-fluorescent and membrane-permeant allowing it to enter the cells before being cleaved by intracellular esterase, forming the membrane-impermeant fluorophore calcein. This fluorescent calcein is retained in the cytoplasm of live cells and thus marks the intracellular space. Calcein is usually evenly distributed throughout the cell and is largely unaffected by either intracellular environment fluctuations or cellular auto-fluorescence. Calcein has been used previously in other volume measurement experiments [72], [73], [74], [75]. Vybrant™ DiI cell-labelling solution (cat# V-22885, Invitrogen Molecular Probes, Eugene, OR) is used as a lipophilic membrane marker [76] added directly to phenol free RPMI to uniformly label cell suspensions. DiI has been used for measuring cellular volume in various cell types including glioma cells [77]. Molecular Probes' Hoechst 33342 trihydrochloride, tryhydrate nucleic acid stain (cat#H1399, Invitrogen Molecular Probes Inc, Eugene, OR) is a cell-permeant nuclear counter stain that emits blue fluorescence when bound to dsDNA and is used as a nuclei marker. Phenylarsine Oxide (PAO) (cat#P3075, sigma, Saint Louis, MO) is an inhibitor of cell surface receptor internalization. Agarose mixture (1% Agarose (Molecular Grade, cat# BIO-41025, Bioline, Luckenwalde, Germany), 0.25% W/V Bovine Serum Albumin in phosphate buffered saline kept at 37°C) was prepared for use as an imaging medium to minimise lateral movements and keep the cells in place during imaging.

### Preparation and Purification of Recombinant Proteins

Recombinant proteins used in this study include β-cateninEE, full-length FLAG-GSK3β, H_6_-m Axin-HA, full-length H_6_-APC-EE and the intracellular domain of E-cadherin. The expression and purification of all recombinant proteins are as described in Text S1.

### Cell Compartment Volume Measurements and Analysis with Confocal 3D Microscopy

Trypsinised cells from the 5 cell cultures was stained with Calcein AM fluorescent dye, Hoechst 33342 nucleic acid stain and Vybrant DiI cell labelling solution to mark the cytoplasm, nuclei and membrane respectively. The stained cells were embedded evenly in agarose mixture within a Sykes Moore Chamber. 3D image stacks of the embedded cells were acquired using the Olympus FV1000 confocal microscope and processed using Metamorph Premier image processing software. Image analysis and cell compartment calculations were performed in Matlab [78] to obtain the resultant volumetric calculations. Details of the protocol can be obtained from Text S2 and Figures S1, S2, S3, and S4.

### Measuring protein concentrations in mammalian cells

Utilizing the measured volume and quantitative western blot analysis technique, the whole cell protein concentrations of key Wnt proteins β-catenin, GSK3β, Axin and APC as well as the cellular adhesion protein E-cadherin for the five mammalian cell lines HEK293T, MDCK, Caco-2, SW480 and SW480APC were systematically quantified and analysed. The compartment concentrations of β-catenin (both total and active forms) were further quantified for the cell lines. Details of the measurement procedures can be found in Text S3 and Figures S5, S6, S7, S8, S9, and S10.

The dependency of the sub-cellular distribution of β-catenin (both total and active forms) on the cell culture conditions for SW480 and SW480APC cells was investigated using confocal imaging. The relative 3D compartment quantification of β-catenin in SW480 and SW480APC was conducted as per described in the Text S3.

### Computational Analysis of the Wnt signalling pathway

The response of Lee *et al.* 2003 [32] model to the protein concentration values associated with the mammalian cytosolic extract system was investigated by employing the full “ODE” (ordinary differential equation) model by Lee *et al.* 2003 [32]. In this study, the association of Lee *et al.*'s model in the context of mammalian whole cell system was investigated by integrating the newly acquired quantitative data (total protein concentrations).

The computational model utilised in the study is a reconstruction of Lee *et al*.'s model [32]. Instead of reducing the model using approximations such as fast binding reactions [32], the complete model comprising all of the rate equations representing all of the interacting components of the pathway were employed. This strategy was chosen as it was unclear if Lee *et al.*'s assumptions for model reduction would hold true for the mammalian systems. Therefore a non-presumptive approach with all the reactions was used. In this integration analysis, Lee *et al*. model was solved in MATLAB [78] using the stiff ODE solver ‘ode23s’ [79]. The stiff ODE solver is commonly used for biochemical reactions due to the potential rapid variations in the solutions caused by differences in the mass-action rate equations. The model was first verified by reproducing the numbers appearing in the Lee *et al*. study using the Xenopus data.

The experimental WCL concentrations measured are total concentration for each individual protein, without information of the distribution of these proteins among the various complexes. The first step in the computational analysis then involves steady state redistribution of the proteins among its complexes (see Figure 10). This analysis involves two phases, both steady state redistribution of proteins among the complexes but with different conditions. Phase A is conducted as a closed system, where all input and output fluxes are switched off. Using the calculated results from Phase A as an initial condition, Phase B repeats the redistribution in an open system (all reactions employed). If the Lee *et al*. model is appropriate for mammalian systems, it is expected the total protein level are to be maintained after redistribution in an open system.

To determine if Lee *et al*. model is steady state representative of Wnt signalling in mammalian cells, steady state simulation (Phase A and B) are conducted with [32] the new total protein concentrations quantitative data and the original set of reaction rates (including fluxes relating to protein production etc) used by Lee *et al*. Specifically, Lee *et al*. model is simulated without Wnt stimulation (Wnt = 0) and starting from the initial concentrations (given by the respective total protein concentrations of the key components in both Lee *et al*.'s and the WCL concentrations). The system ODEs were solved to obtain the new steady states with proteins redistributing among their various complexes initially in a closed system (Phase A), followed by an open system (Phase B) [32]. To test the uniqueness of the final solution for the set of initial concentrations, the computed protein redistribution simulation was also performed via an alternate pathway, namely, using only Phase B and the initial experimental concentrations to obtain the corresponding final steady state solution (open system). The computed open-system steady-state distributions were found to be pathway independent concentrations, suggesting the computed open-system steady states are robust.

## Supporting Information

Figure S1 **Steps for image stack analysis and selection of cells using metamorph.** Fluorescence labelled cytosol (Calcein AM in green), membrane (Vybrant DiI in red) and nuclei (Hoechst 33342 in blue).
(TIF)

Figure S2 **Procedures for image processing and quantification (Steps 1 to 3).** Import, separate channels and segment image. Step 1, IMPORT: Import image stack TIFF for each individual selected cell. Step 2, CHANNEL SEPARATION: Separate independent channel information. Step 3, IMAGE SEGMENTATION: Threshold, filter and fill holes to generate binary masks.
(TIF)

Figure S3 **Procedures for image processing and quantification (Steps 4 and 5).** 3D object filtering, processing and quantification.
(TIF)

Figure S4 **Procedures for image processing and quantification (Steps 6 to 8).** Data consolidation, cell population analysis and volume distribution analysis.
(TIF)

Figure S5 **Western blots for quantitative analysis of whole cell lysates (WCL) for Wnt signalling proteins.** (A) β-catenin (B) APC (C) Axin (D) GSK3β (E) E-cadherin in the different cell lines with recombinant proteins as standards.
(TIF)

Figure S6 **Procedures for quantifying levels of proteins in WCL.** This figure uses β-catenin as the specific protein investigated. Mass of total protein, *TP* per lane (A) calculated based on known amount of protein loaded. The total mass of *specific protein* (*P*) per lane (B) was calculated using the standard curve of known amounts of the *P* in the same western blot. Mass of *P* per ng *TP* loaded (C) calculated by dividing B with A. P per ng TP (D) calculated by averaging n independent sets of C. Average TP per cell (E) acquired from cell count and BCA assay experiments and used to calculate average P per cell (F) by multiplying D with E. Relative molecular weight of *P,* (M_r_) used to calculate nanomole of P per cell, dividing F with M_r_. Whole cell volume of a resting cell (H) was measured in this study and used to calculate the Molar concentration of *P* per cell (I), dividing G with H. Final concentration of P per cell calculated in nM per cell (J).
(TIF)

Figure S7 **Western blots for quantitative compartment analysis of β-catenin.** β-catenin levels in (A) HEK293T, (B) Caco-2, (C) MDCK, (D) SW480 and (E) SW480APC with recombinant β-catenin as protein standards.
(TIF)

Figure S8 **Western blots for quantitative compartment analysis of “active” β-catenin.** (A) Western blot of two identical sets of recombinant β-catenin probed for total or “active” β-catenin. The standard curves generated by these two sets were used for correlating protein levels detected by the two antibodies. “Active” β-catenin levels in (B) HEK293T, (C) Caco-2, (D) MDCK, (E) SW480 and (F) SW480APC with recombinant β-catenin as protein standards.
(TIF)

Figure S9 **Procedures for quantifying proteins levels for sub-cellular fractions.** The mass of β-catenin per lane (E) from the western blot was calculated using known amounts of recombinant β-catenin. Mass of β-catenin in the loading sample (F) and subsequently total cell volume (G) was calculated by scaling to the original cell volume (B). Dividing F by total cell count (A) gives the mass of β-catenin per cell (H). Relative molecular weight of β-catenin (M_r_) was then used to calculate nanomole of β-catenin per cell (I = H÷M_r_). Whole cell volume of a resting cell (J) was acquired in this study and used to calculate the molar concentration of β-catenin per cell (K = I÷J). Final concentration of β-catenin per cell calculated in nM (L).
(TIF)

Figure S10 **Dependency of β-catenin distribution in SW480 and SW480APC on confluency.** 3D confocal imaging and compartment analysis at different confluency: (A) SW480 and (B) SW480APC at low confluency; (C) SW480 and (D) SW480APC at high confluency. Panels show the (i) overlay (DAPI in blue and β-catenin in green), (ii) DAPI, (iii) β-catenin, (iv) nuclear β-catenin intensity and (v) non-nuclear β-catenin intensity images. (E) Results of compartmental analysis in intensity per voxel shows higher nuclear∶non-nuclear β-catenin ratio (>1) for low confluency samples. Lower nuclear∶non-nuclear β-catenin ratio (<1) is observed for highly confluent samples. (F) Sub-cellular fractionation results for both cell-lines at difference confluency in β-catenin concentrations (nM). Increasing cytosolic and membrane β-catenin concentrations are observed with increasing confluency with the nuclear β-catenin levels remaining constant. (G and H) β-catenin compartment distribution (in % of compartment summation), nuclear β-catenin distribution decreases and the cytosolic β-catenin level increases with increasing confluency. The membrane levels remained constant. Note: panels ii–v are grayscaled for visual clarity.
(TIF)

Figure S11 **Sub-cellular distribution of “active” β-catenin in mammalian cells.** (A) Compartment concentrations of “active” β-catenin in HEK293T, Caco-2, MDCK, SW480 and SW480APC cells. (B) Percentage distribution of “active” β-catenin for the five cell lines.
(TIF)

Figure S12 **Cell population sizing and selection (HEK293T).** Population statistics of HEK293T, showing the (A) histograms of normalised volume (Calcein AM/Hoechst); (B) Scatter plot of fluorescent marked volumes (Calcein AM vs. Hoechst) showing a good linear relationship; (C) Scatter plot of fluorescent marked volumes (Calcein AM vs. Hoechst) showing population isolation and the cut-off volumes applied.
(TIF)

Figure S13 **Cell population sizing and selection (MDCK).** Population statistics of MDCK, showing the (A) histograms of normalised volume (Calcein AM/Hoechst); (B) Scatter plot of fluorescent marked volumes (Calcein AM vs. Hoechst) showing a good linear relationship; (C) Scatter plot of fluorescent marked volumes (Calcein AM vs. Hoechst) showing population isolation and the cut-off volumes applied.
(TIF)

Figure S14 **Cell population sizing and selection (Caco-2).** Population statistics of Caco-2, showing the (A) histograms of normalised volume (Calcein AM/Hoechst); (B) Scatter plot of fluorescent marked volumes (Calcein AM vs. Hoechst) showing a good linear relationship; (C) Scatter plot of fluorescent marked volumes (Calcein AM vs. Hoechst) showing population isolation and the cut-off volumes applied.
(TIF)

Figure S15 **Cell population sizing and selection (SW480).** Population statistics of SW480, showing the (A) histograms of normalised volume (Calcein AM/Hoechst); (B) Scatter plot of fluorescent marked volumes (Calcein AM vs. Hoechst) showing a good linear relationship; (C) Scatter plot of fluorescent marked volumes (Calcein AM vs. Hoechst) showing population isolation and the cut-off volumes applied.
(TIF)

Text S1 **Preparation and Purification of Recombinant Proteins.**
(DOC)

Text S2 **Protocol for confocal cell compartment volume measurements and analysis.**
(DOC)

Text S3 **Measurement Protocol for Protein Concentrations in Mammalian Cells.**
(DOC)

## References

[1] Coudreuse D, Korswagen HC (2007). The making of Wnt: new insights into Wnt maturation, sorting and secretion.. Development.

[2] Nelson WJ, Nusse R (2004). Convergence of Wnt, β-Catenin, and Cadherin Pathways.. Science.

[3] Logan CY, Nusse R (2004). The Wnt Signaling Pathway in Development and Disease.. Annual Review of Cell and Developmental Biology.

[4] Moon RT, Kohn AD, Ferrari GVD, Kaykas A (2004). WNT and β-catenin signalling: diseases and therapies.. Nat Rev Genet.

[5] Polakis P (2000). Wnt signaling and cancer.. Genes & Development.

[6] Clevers H (2006). Wnt/β-catenin signaling in development and disease.. Cell.

[7] Harris TJC, Peifer M (2005). Decisions, decisions: β-catenin chooses between adhesion and transcription.. Trends in Cell Biology.

[8] Kishida S, Yamamoto H, Ikeda S, Kishida M, Sakamoto I (1998). Axin, a Negative Regulator of the Wnt Signaling Pathway, Directly Interacts with Adenomatous Polyposis Coli and Regulates the Stabilization of β-Catenin.. J Biol Chem.

[9] Behrens J, Jerchow B-A, Würtele M, Grimm J, Asbrand C (1998). Functional Interaction of an Axin Homolog, Conductin, with β-Catenin, APC, and GSK3β.. Science.

[10] Liu C, Yiming L, Semenov M, Han C, Baeg G-H (2002). Control of β-catenin phosphorylation/degradation by a dual-kinase mechanism.. Cell.

[11] Aberle H, Bauer A, Stappert J, Kispert A, Kemler R (1997). β-catenin is a target for the ubiquitin-proteosome pathway.. The EMBO Journal.

[12] Kimelman D, Xu W (2006). β-Catenin destruction complex: insights and questions from a structural perspective.. Oncogene.

[13] Xing Y, Clements WK, Le Trong I, Hinds TR, Stenkamp R (2004). Crystal Structure of a β-Catenin/APC Complex Reveals a Critical Role for APC Phosphorylation in APC Function.. Molecular Cell.

[14] Ha N-C, Tonozuka T, Stamos JL, Choi H-J, Weis WI (2004). Mechanism of Phosphorylation-Dependent Binding of APC to β-Catenin and Its Role in β-Catenin Degradation.. Molecular Cell.

[15] Behrens J, von Kries JP, Kuhl M, Bruhn L, Wedlich D (1996). Functional interaction of β-catenin with the transcription factor LEF-1.. Nature.

[16] Luo W, Lin SC (2004). Axin: A Master Scaffold for Multiple Signaling Pathways.. Neurosignals.

[17] Furuhashi M, Yagi K, Yamamoto H, Furukawa Y, Shimada S (2001). Axin Facilitates Smad3 Activation in the Transforming Growth Factor β Signaling Pathway.. Mol Cell Biol.

[18] Zhang Y, Neo SY, Wang X, Han J, Lin S-C (1999). Axin Forms a Complex with MEKK1 and Activates c-Jun NH2-terminal Kinase/Stress-activated Protein Kinase through Domains Distinct from Wnt Signaling.. Journal of Biological Chemistry.

[19] Faux MC, Coates JL, Catimel B, Cody S, Clayton AHA (2008). Recruitment of adenomatous polyposis coli and β-catenin to axin-puncta.. Oncogene.

[20] Näthke IS, Adams CL, Polakis P, Sellin JH, Nelson WJ (1996). The Adenomatous Polyposis Coli Tumor Suppressor Protein Localizes to Plasma Membrane Sites Involved in Active Cell Migration.. The Journal of Cell Biology.

[21] Rosin-Arbesfeld R, Ihrke G, Bienz M (2001). Actin-dependent membrane association of the APC tumour suppressor in polarized mammalian epithelial cells.. The EMBO Journal.

[22] Kaplan KB, Burds AA, Swedlow JR, Bekir SS, Sorger PK (2001). A role for the Adenomatous Polyposis Coli protein in chromosome segregation.. Nat Cell Biol.

[23] Bienz M (2002). The subcellular destinations of apc proteins.. Nat Rev Mol Cell Biol.

[24] Gumbiner BM (2000). Regulation of Cadherin Adhesive Activity.. J Cell Biol.

[25] Behrens J (1999). Cadherins and Catenins: Role in Signal Transduction and Tumor Progression.. Cancer and Metastasis Reviews.

[26] Christofori G (2003). Changing neighbours, changing behaviour: cell adhesion molecule-mediated signalling during tumour progression.. The EMBO Journal.

[27] Giles RH, van Es JH, Clevers H (2003). Caught up in a Wnt storm: Wnt signaling in cancer.. Biochimica et Biophysica Acta (BBA) - Reviews on Cancer.

[28] Ilyas M, Straub J, Tomlinson IPM, Bodmer WF (1999). Genetic pathways in colorectal and other cancers.. European Journal of Cancer.

[29] Kitano H (2002). Systems Biology: A Brief Overview.. Science.

[30] Vayttaden SJ, Ajay SM, Bhalla US (2004). A Spectrum of Models of Signaling Pathways.. ChemBioChem.

[31] Ideker T, Galitski T, Hood L (2001). A New Approach to Decoding Life: Systems Biology.. Annual Review of Genomics and Human Genetics.

[32] Lee E, Salic A, Kruger R, Heinrich R, Kirschner MW (2003). The Roles of APC and Axin Derived from Experimental and Theoretical Analysis of the Wnt Pathway.. PLoS Biology.

[33] Kim D, Rath O, Kolch W, Cho K (2007). A hidden oncogenic positive feedback loop caused by crosstalk between Wnt and ERK Pathways.. Oncogene.

[34] Sun Y-C (2009). Examination of effects of GSK3β phosphorylation, β-catenin phosphorylation, and β-catenin degradation on kinetics of Wnt signaling pathway using computational method.. Theoretical Biology and Medical Modelling.

[35] Wawra C, Kuhl M, Kestler HA (2007). Extended analyses of the Wnt/β-catenin pathway: Robustness and oscillatory behaviour.. FEBS Letters.

[36] Cho K-H, Baek S, Sung M-H (2006). Wnt pathway mutations selected by optimal β-catenin signaling for tumorigenesis.. FEBS Lett.

[37] Graham FL, Smiley J, Russell WC, Nairn R (1977). Characteristics of a Human Cell Line Transformed by DNA from Human Adenovirus Type 5.. Journal of General Virology.

[38] Leighton J, Brada Z, Estes LW, Justh G (1969). Secretory Activity and Oncogenicity of a Cell Line (MDCK) Derived from Canine Kidney.. Science.

[39] Gaush CR, Hard WL, Smith TF (1966). Characterization of an established line of canine kidney cells (MDCK).. Proceedings of the Society for Experimental Biology and Medicine.

[40] Fogh J, Wright WC, Loveless JD (1977). Absence of HeLa cell contamination in 169 cell lines derived from human tumors.. Journal of the National Cancer Institute.

[41] Leibovitz A, Stinson JC, McCombs WB, McCoy CE, Mazur KC (1976). Classification of Human Colorectal Adenocarcinoma Cell Lines.. Cancer Research.

[42] Faux MC, Ross JL, Meeker C, Johns T, Ji H (2004). Restoration of full-length adenomatous polyposis coli (APC) protein in a colon cancer cell line enhances cell adhesion.. J Cell Sci.

[43] Gumbiner BM (1996). Cell Adhesion: The Molecular Basis of Tissue Architecture and Morphogenesis.. Cell.

[44] Takeichi M (1990). Cadherins: A Molecular Family Important in Selective Cell-Cell Adhesion.. Annual Review of Biochemistry.

[45] Fantes PA, Grant WD, Pritchard RH, Sudbery PE, Wheals AE (1975). The regulation of cell size and the control of mitosis.. Journal of Theoretical Biology.

[46] Migita S, Funakoshi K, Tsuya D, Yamazaki T, Taniguchi A (2010). Cell cycle and size sorting of mammalian cells using a microfluidic device.. Analytical Methods.

[47] Ycas M, Sugita M, Bensam A (1965). A model of cell size regulation.. Journal of Theoretical Biology.

[48] Roy G, Sauvé R (1987). Effect of anisotonic media on volume, ion and amino-acid content and membrane potential of kidney cells (MDCK) in culture.. Journal of Membrane Biology.

[49] Nishisho I, Nakamura Y, Miyoshi Y, Miki Y, Ando H (1991). Mutations of Chromosome 5q21 Genes in FAP and Colorectal Cancer Patients.. Science.

[50] Hogan C, Serpente N, Cogram P, Hosking CR, Bialucha CU (2004). Rap1 Regulates the Formation of E-Cadherin-Based Cell-Cell Contacts.. Mol Cell Biol.

[51] Suyama K, Shapiro I, Guttman M, Hazan RB (2002). A signaling pathway leading to metastasis is controlled by N-cadherin and the FGF receptor.. Cancer Cell.

[52] Staal FJT, van Noort M, Strous GJ, Clevers HC (2002). Wnt signals are transmitted through N-terminally dephosphorylated β-catenin.. EMBO J.

[53] van Noort M, Meeldijk J, van der Zee R, Destree O, Clevers H (2002). Wnt Signaling Controls the Phosphorylation Status of β-Catenin.. Journal of Biological Chemistry.

[54] Mariadason JM, Bordonaro M, Aslam F, Shi L, Kuraguchi M (2001). Down-Regulation of β-Catenin TCF Signaling Is Linked to Colonic Epithelial Cell Differentiation.. Cancer research.

[55] Rosin-Arbesfeld R, Cliffe A, Brabletz T, Bienz M (2003). Nuclear export of the APC tumour suppressor controls β-catenin function in transcription.. EMBO J.

[56] Fearnhead NS, Britton MP, Bodmer WF (2001). The ABC of APC.. Hum Mol Genet.

[57] Rowan AJ, Lamlum H, Ilyas M, Wheeler J, Straub J (2000). APC mutations in sporadic colorectal tumors: A mutational “hotspot” and interdependence of the “two hits”.. Proceedings of the National Academy of Sciences.

[58] Smith KJ, Johnson KA, Bryan TM, Hill DE, Markowitz S (1993). The APC gene product in normal and tumor cells.. Proceedings of the National Academy of Sciences of the United States of America.

[59] Fodde R, Smits R, Clevers H (2001). APC, Signal transduction and genetic instability in colorectal cancer.. Nat Rev Cancer.

[60] Schwarz-Romond T, Merrifield C, Nichols BJ, Bienz M (2005). The Wnt signalling effector Dishevelled forms dynamic protein assemblies rather than stable associations with cytoplasmic vesicles.. J Cell Sci.

[61] Schwarz-Romond T, Metcalfe C, Bienz M (2007). Dynamic recruitment of axin by Dishevelled protein assemblies.. J Cell Sci.

[62] MacDonald BT, Tamai K, He X (2009). Wnt/β-Catenin Signaling: Components, Mechanisms, and Diseases.. Developmental Cell.

[63] van Leeuwen IMM, Byrne HM, Jensen OE, King JR (2007). Elucidating the interactions between the adhesive and transcriptional functions of β-catenin in normal and cancerous cells.. Journal of Theoretical Biology.

[64] van Leeuwen IMM, Mirams GR, Walter A, Fletcher A, Murray P (2009). An integrative computational model for intestinal tissue renewal.. Cell Proliferation.

[65] Taelman VF, Dobrowolski R, Plouhinec J-L, Fuentealba LC, Vorwald PP (2010). Wnt Signaling Requires Sequestration of Glycogen Synthase Kinase 3 inside Multivesicular Endosomes.. Cell.

[66] Schwarz-Romond T, Fiedler M, Shibata N, Butler P, Kikuchi A (2007). The DIX domain of Dishevelled confers Wnt signaling by dynamic polymerization.. Nat Struct Mol Biol.

[67] Schwarz-Romond T, Merrifield C, Nichols B, Bienz M (2005). The Wnt signalling effector Dishevelled forms dynamic protein assemblies rather than stable associations with cytoplasmic vesicles.. J Cell Sci.

[68] Penman GA, Leung L, Nathke IS (2005). The adenomatous polyposis coli protein (APC) exists in two distinct soluble complexes with different functions.. J Cell Sci.

[69] Graham FL, Smiley J, Russell WC, Nairn R (1977). Characteristics of a Human Cell Line Transformed by DNA from Human Adenovirus Type 5.. J Gen Virol.

[70] Ridley A, Comoglio P, Hall A (1995). Regulation of scatter factor/hepatocyte growth factor responses by Ras, Rac, and Rho in MDCK cells.. Mol Cell Biol.

[71] Leibovitz A, Stinson JC, McCombs WB, McCoy CE, Mazur KC (1976). Classification of Human Colorectal Adenocarcinoma Cell Lines.. Cancer Res.

[72] Bush PG, Hodkinson PD, Hamilton GL, Hall AC (2005). Viability and volume of in situ bovine articular chondrocytes–changes following a single impact and effects of medium osmolarity.. Osteoarthritis and Cartilage.

[73] Chacon E, Reece JM, Nieminen AL, Zahrebelski G, Herman B (1994). Distribution of electrical potential, pH, free Ca2+, and volume inside cultured adult rabbit cardiac myocytes during chemical hypoxia: a multiparameter digitized confocal microscopic study.. Biophysical Journal.

[74] Farinas J, Simanek V, Verkman AS (1995). Cell volume measured by total internal reflection microfluorimetry: application to water and solute transport in cells transfected with water channel homologs.. Biophysical Journal.

[75] Poole C, Brookes N, Clover G (1993). Keratocyte networks visualised in the living cornea using vital dyes.. J Cell Sci.

[76] Veranic P, Lokar M, Schütz GJ, Weghuber J, Wieser S (2008). Different Types of Cell-to-Cell Connections Mediated by Nanotubular Structures.. Biophysical Journal.

[77] Habela CW, Sontheimer H (2007). Cytoplasmic Volume Condensation Is an Integral Part of Mitosis.. Cell cycle.

[78] Mathworks (2007). MATLAB..

[79] Shampine LF, Reichelt MW (1997). The matlab ode suite.. SIAM journal on scientific computing.

