# Supplementary figures and images for: Wnt Signalling Pathway Parameters for Mammalian Cells
Source: PLoS One. 2012 Feb 21;7(2):e31882. doi: 10.1371/journal.pone.0031882 (PMC3283727; doi:10.1371/journal.pone.0031882)

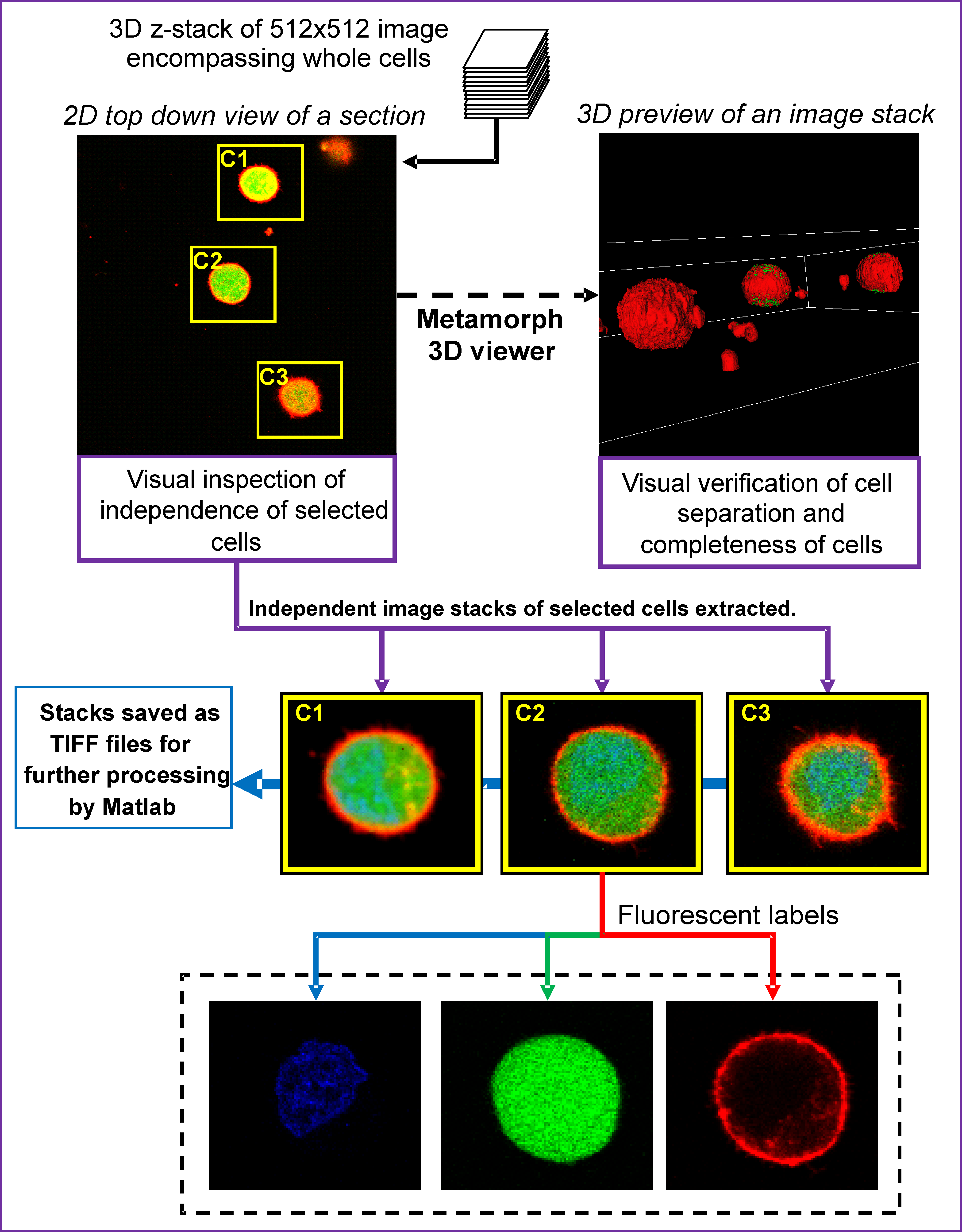

Supplement: Figure S1 — Steps for image stack analysis and selection of cells using metamorph. Fluorescence labelled cytosol (Calcein AM in green), membrane (Vybrant DiI in red) and nuclei (Hoechst 33342 in blue). (TIF) [file pone.0031882.s001.tif]

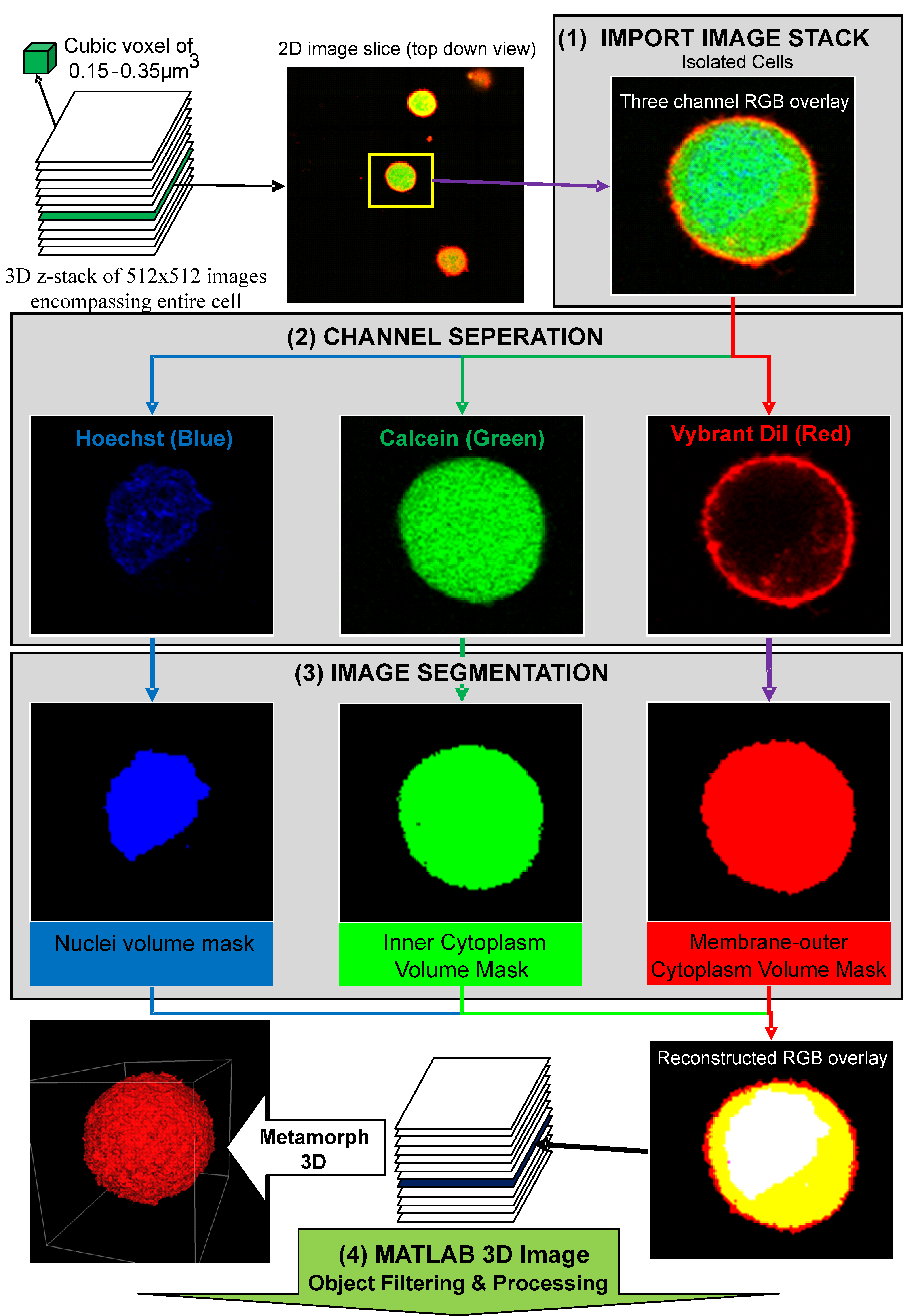

Supplement: Figure S2 — Procedures for image processing and quantification (Steps 1 to 3). Import, separate channels and segment image. Step 1, IMPORT: Import image stack TIFF for each individual selected cell. Step 2, CHANNEL SEPARATION: Separate independent channel information. Step 3, IMAGE SEGMENTATION: Threshold, filter and fill holes to generate binary masks. (TIF) [file pone.0031882.s002.tif]

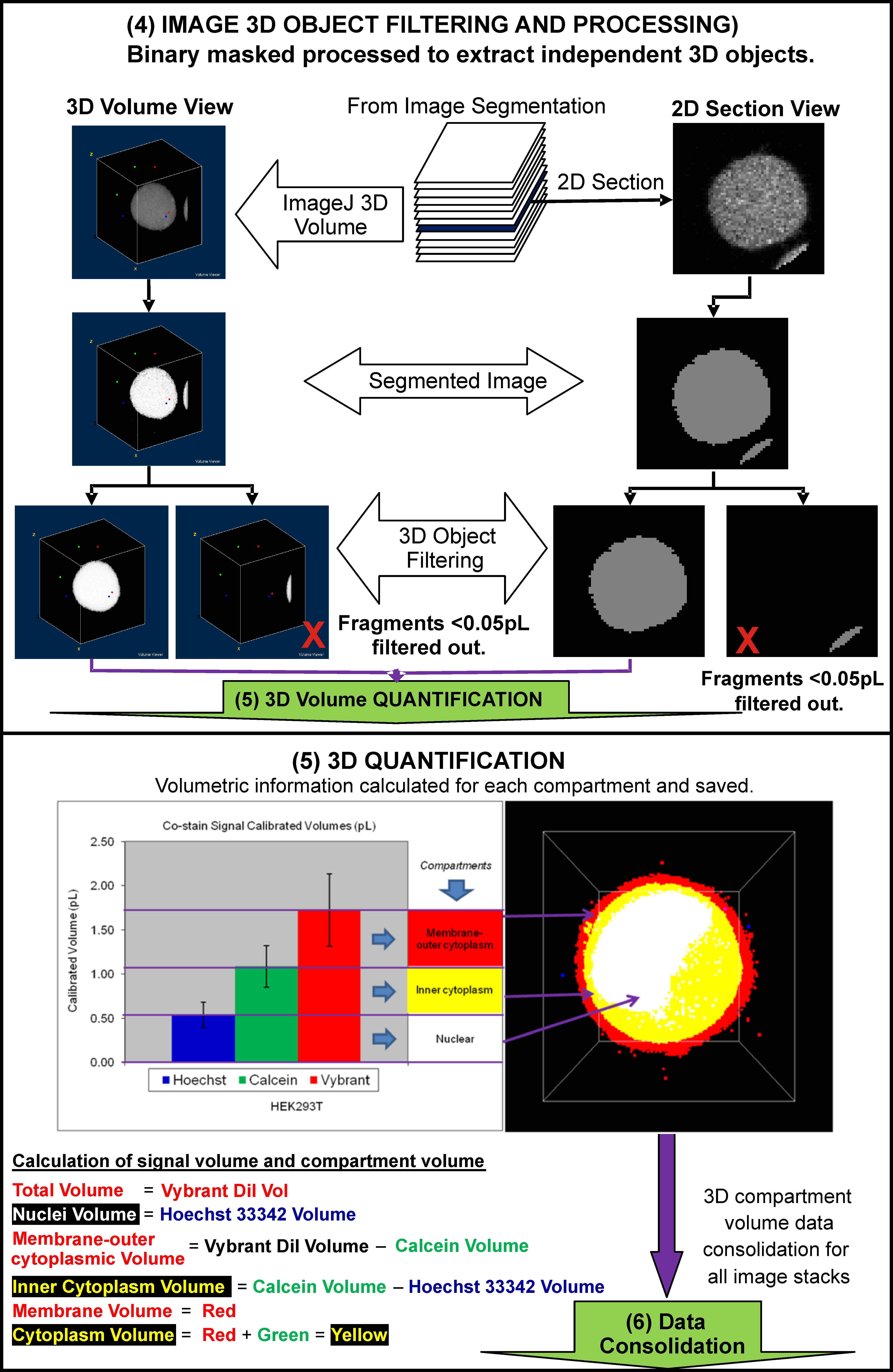

Supplement: Figure S3 — Procedures for image processing and quantification (Steps 4 and 5). 3D object filtering, processing and quantification. (TIF) [file pone.0031882.s003.tif]

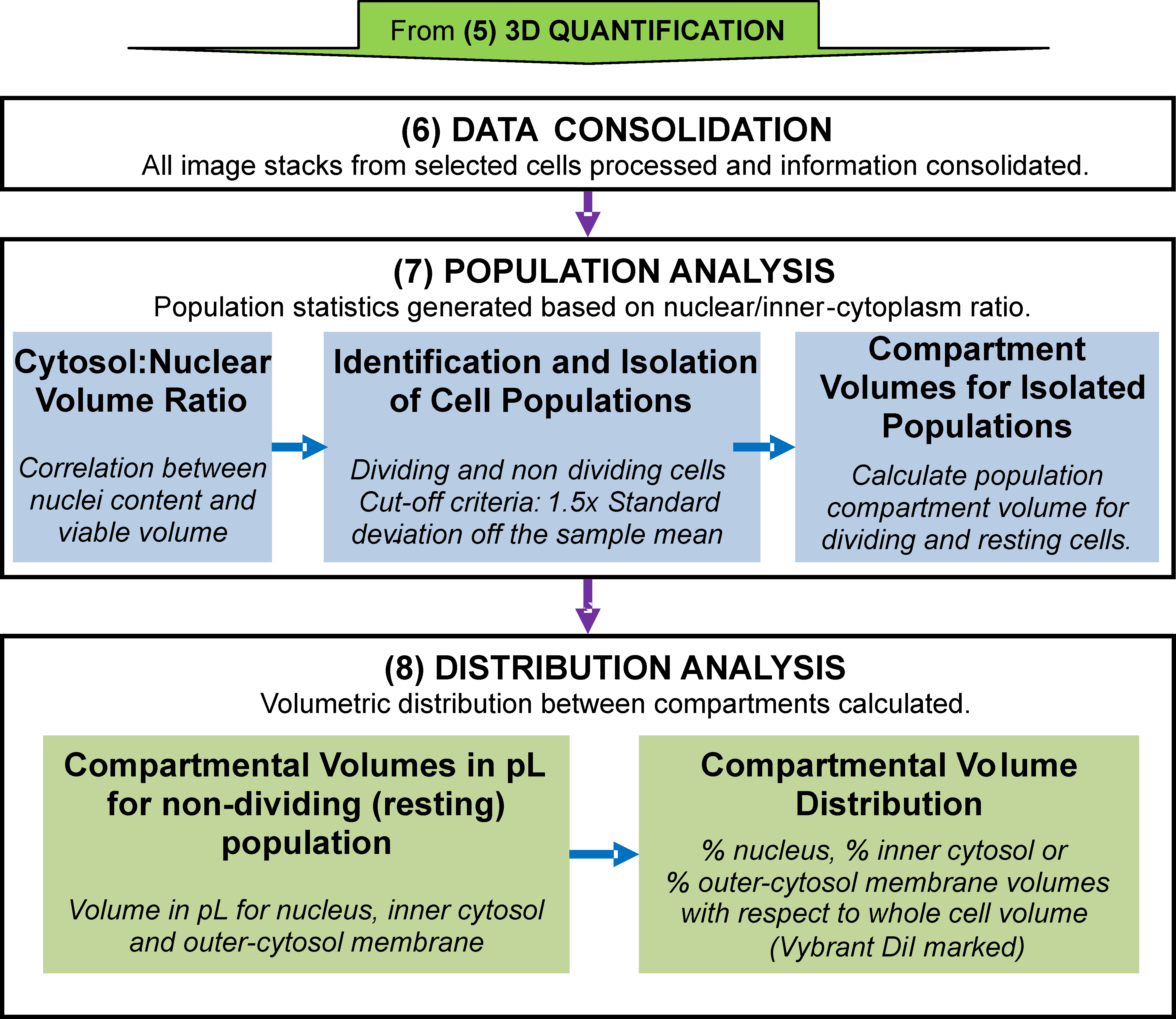

Supplement: Figure S4 — Procedures for image processing and quantification (Steps 6 to 8). Data consolidation, cell population analysis and volume distribution analysis. (TIF) [file pone.0031882.s004.tif]

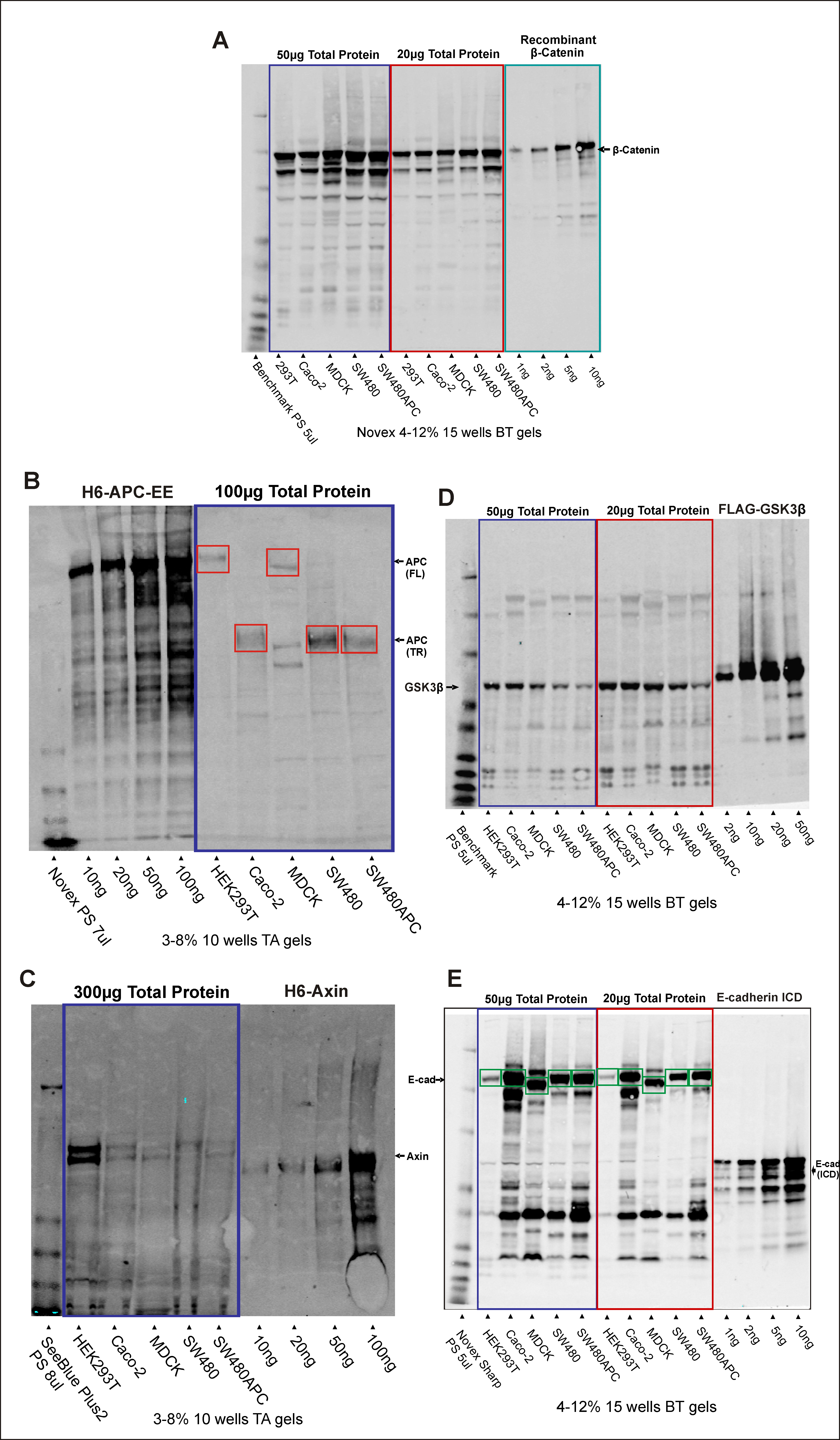

Supplement: Figure S5 — Western blots for quantitative analysis of whole cell lysates (WCL) for Wnt signalling proteins. (A) β-catenin (B) APC (C) Axin (D) GSK3β (E) E-cadherin in the different cell lines with recombinant proteins as standards. (TIF) [file pone.0031882.s005.tif]

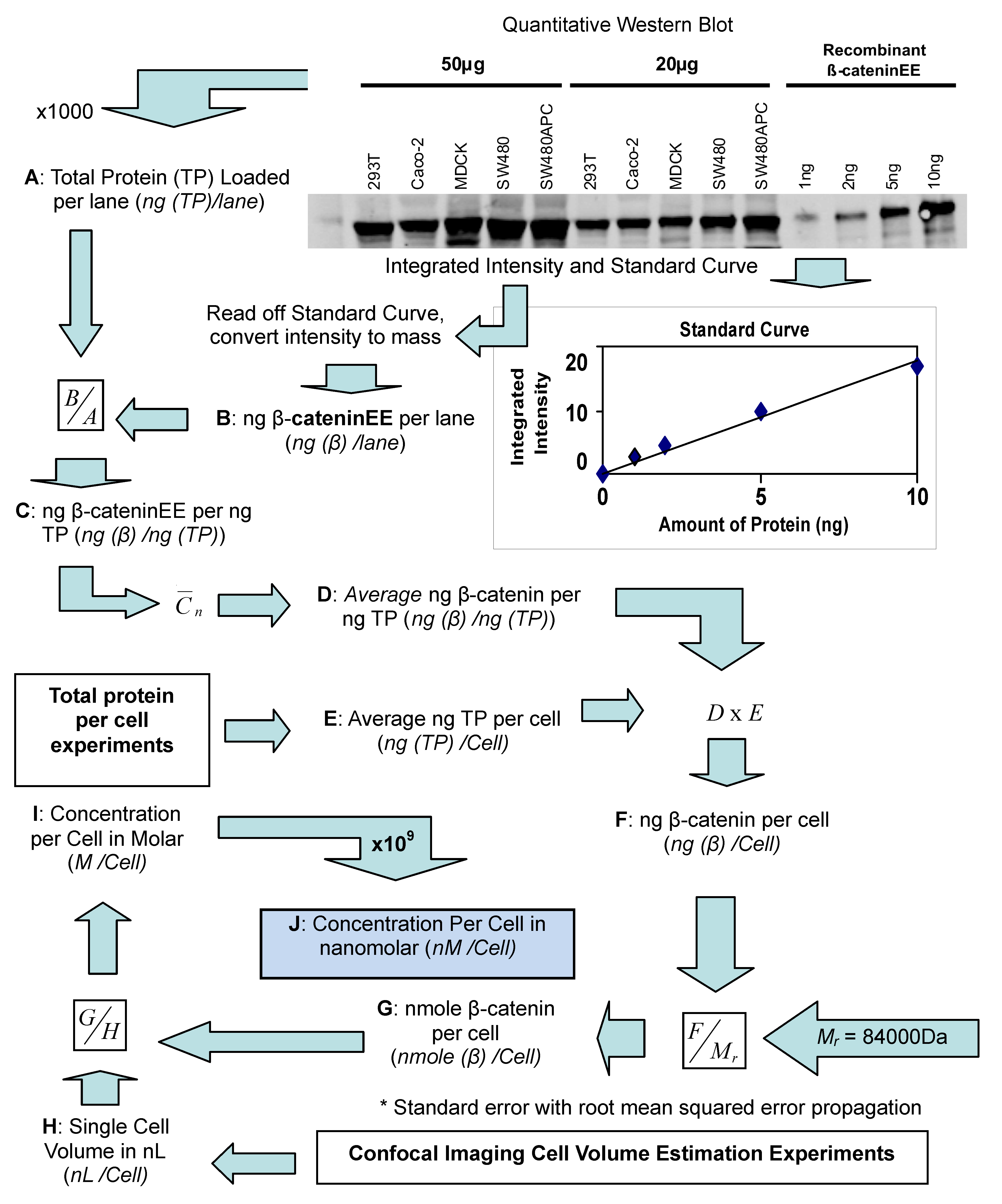

Supplement: Figure S6 — Procedures for quantifying levels of proteins in WCL. This figure uses β-catenin as the specific protein investigated. Mass of total protein, TP per lane (A) calculated based on known amount of protein loaded. The total mass of specific protein (P) per lane (B) was calculated using the standard curve of known amounts of the P in the same western blot. Mass of P per ng TP loaded (C) calculated by dividing B with A. P per ng TP (D) calculated by averaging n independent sets of C. Average TP per cell (E) acquired from cell count and BCA assay experiments and used to calculate average P per cell (F) by multiplying D with E. Relative molecular weight of P, (Mr) used to calculate nanomole of P per cell, dividing F with Mr. Whole cell volume of a resting cell (H) was measured in this study and used to calculate the Molar concentration of P per cell (I), dividing G with H. Final concentration of P per cell calculated in nM per cell (J). (TIF) [file pone.0031882.s006.tif]

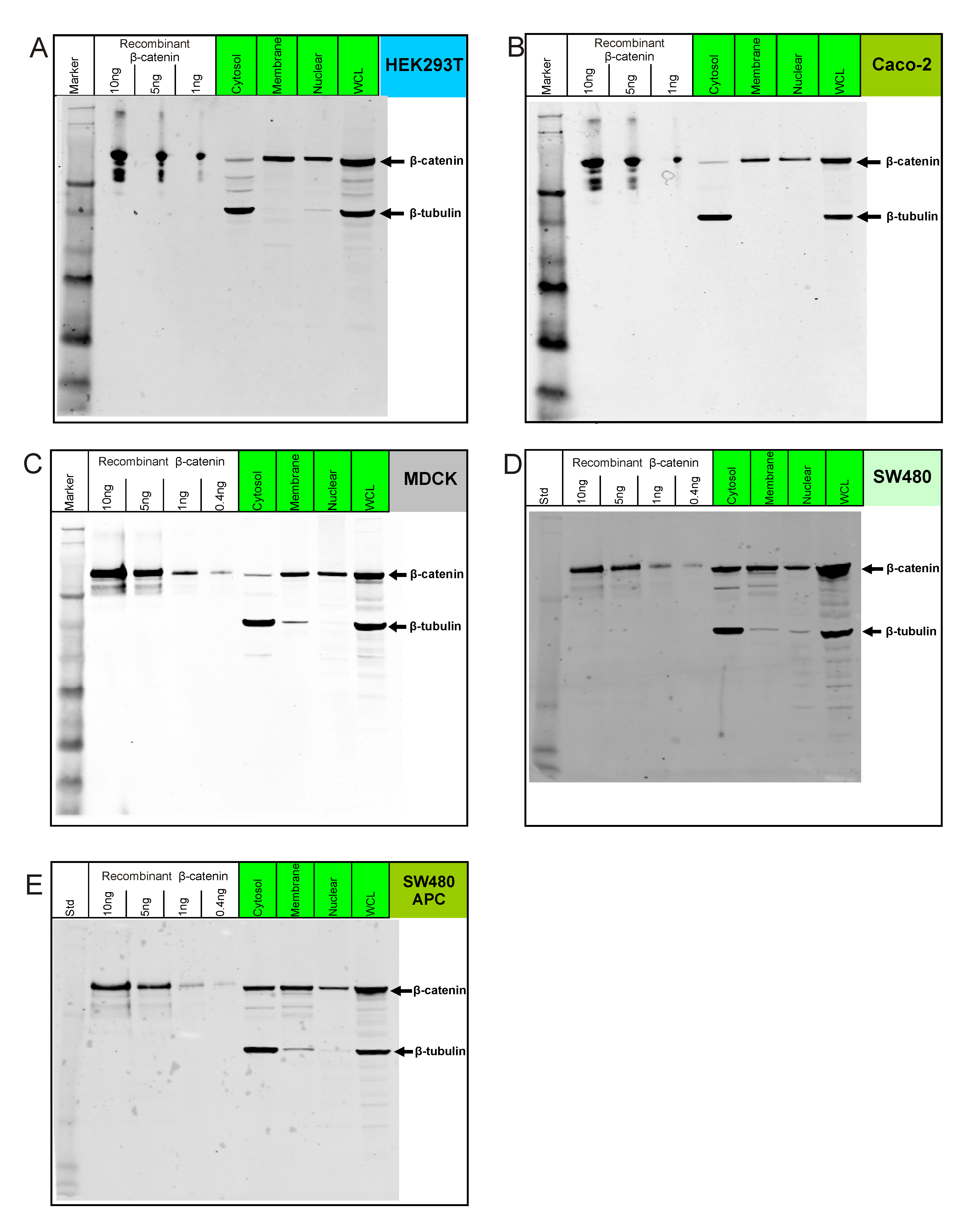

Supplement: Figure S7 — Western blots for quantitative compartment analysis of β-catenin. β-catenin levels in (A) HEK293T, (B) Caco-2, (C) MDCK, (D) SW480 and (E) SW480APC with recombinant β-catenin as protein standards. (TIF) [file pone.0031882.s007.tif]

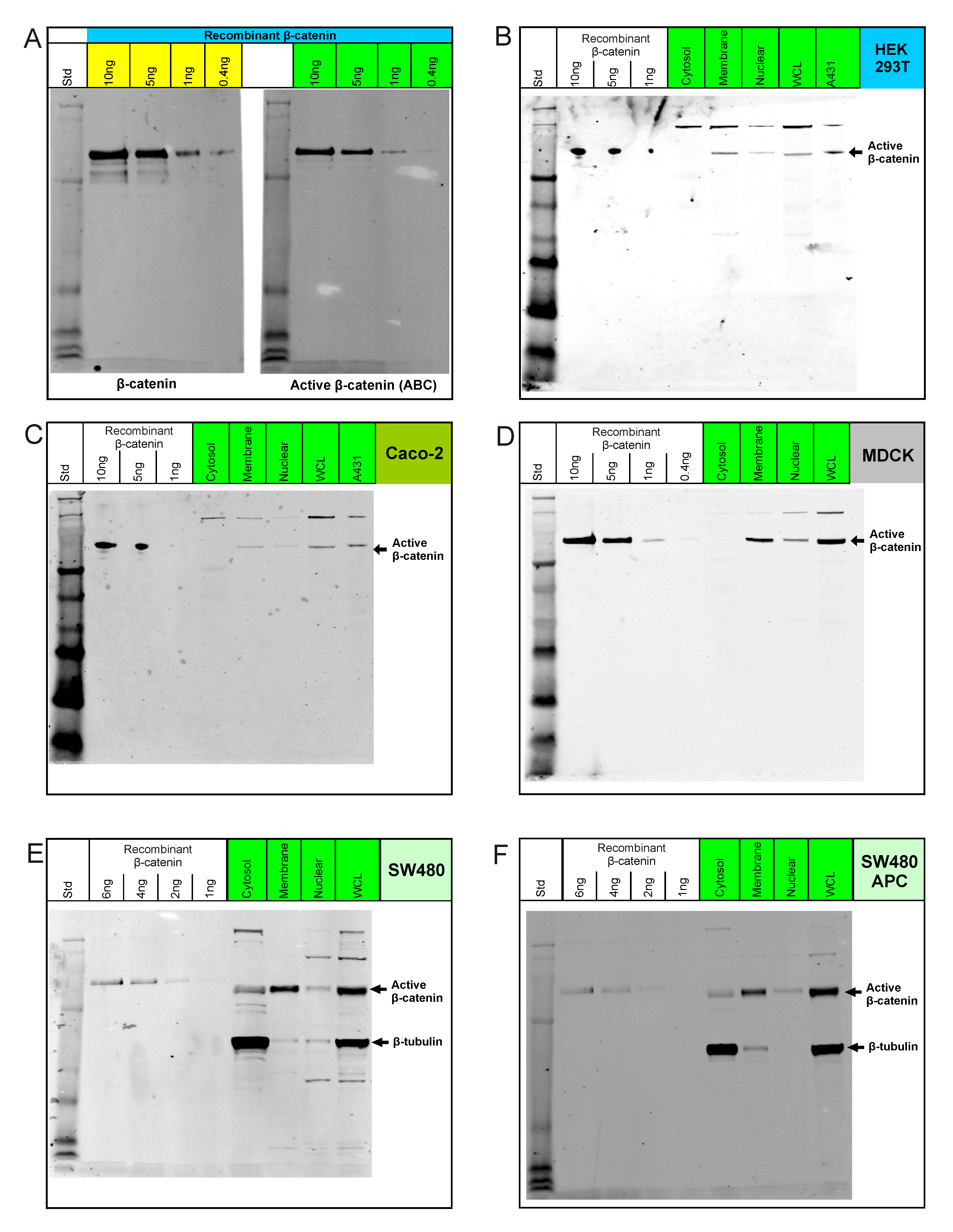

Supplement: Figure S8 — Western blots for quantitative compartment analysis of “active” β-catenin. (A) Western blot of two identical sets of recombinant β-catenin probed for total or “active” β-catenin. The standard curves generated by these two sets were used for correlating protein levels detected by the two antibodies. “Active” β-catenin levels in (B) HEK293T, (C) Caco-2, (D) MDCK, (E) SW480 and (F) SW480APC with recombinant β-catenin as protein standards. (TIF) [file pone.0031882.s008.tif]

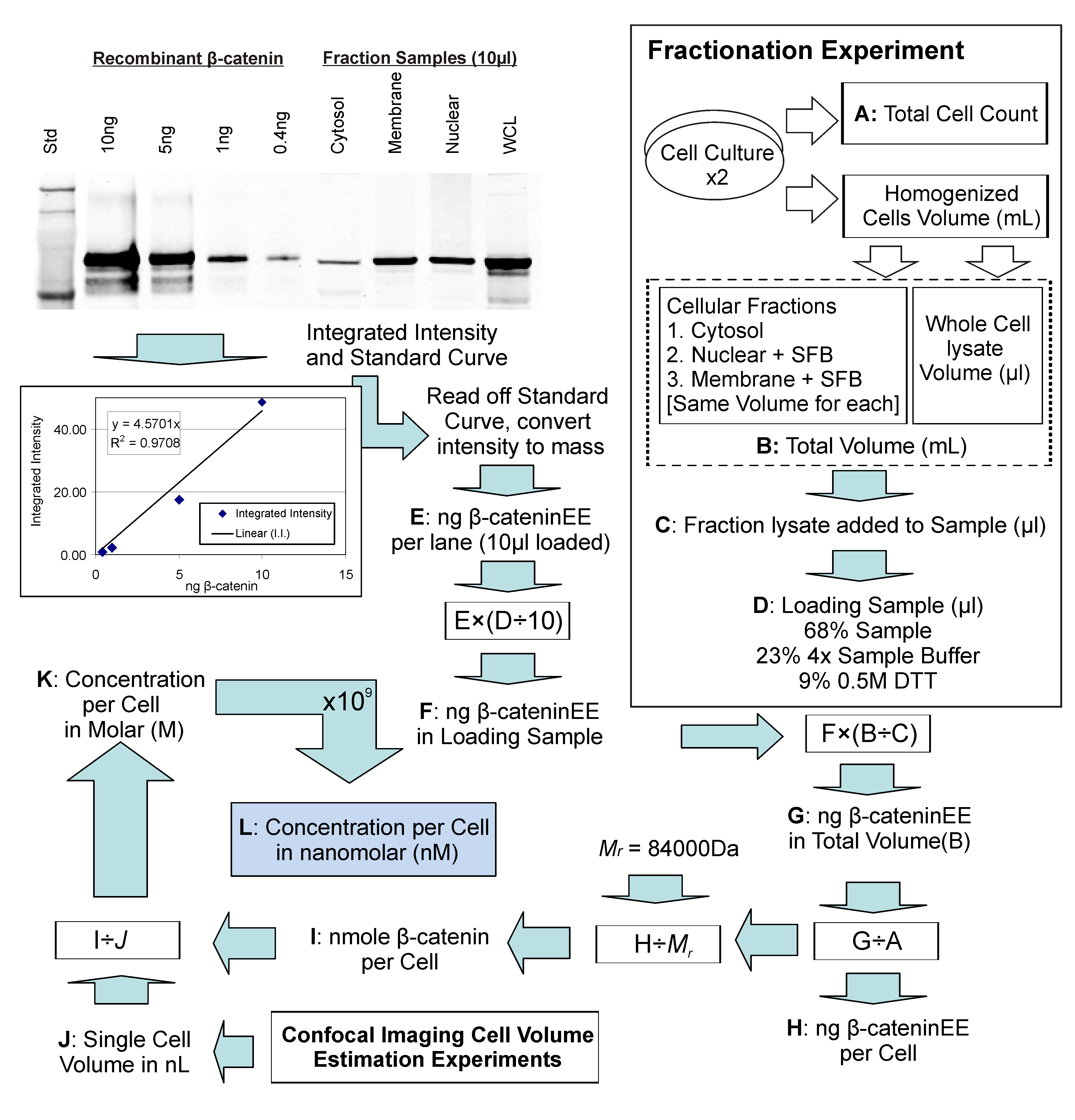

Supplement: Figure S9 — Procedures for quantifying proteins levels for sub-cellular fractions. The mass of β-catenin per lane (E) from the western blot was calculated using known amounts of recombinant β-catenin. Mass of β-catenin in the loading sample (F) and subsequently total cell volume (G) was calculated by scaling to the original cell volume (B). Dividing F by total cell count (A) gives the mass of β-catenin per cell (H). Relative molecular weight of β-catenin (Mr) was then used to calculate nanomole of β-catenin per cell (I = H÷Mr). Whole cell volume of a resting cell (J) was acquired in this study and used to calculate the molar concentration of β-catenin per cell (K = I÷J). Final concentration of β-catenin per cell calculated in nM (L). (TIF) [file pone.0031882.s009.tif]

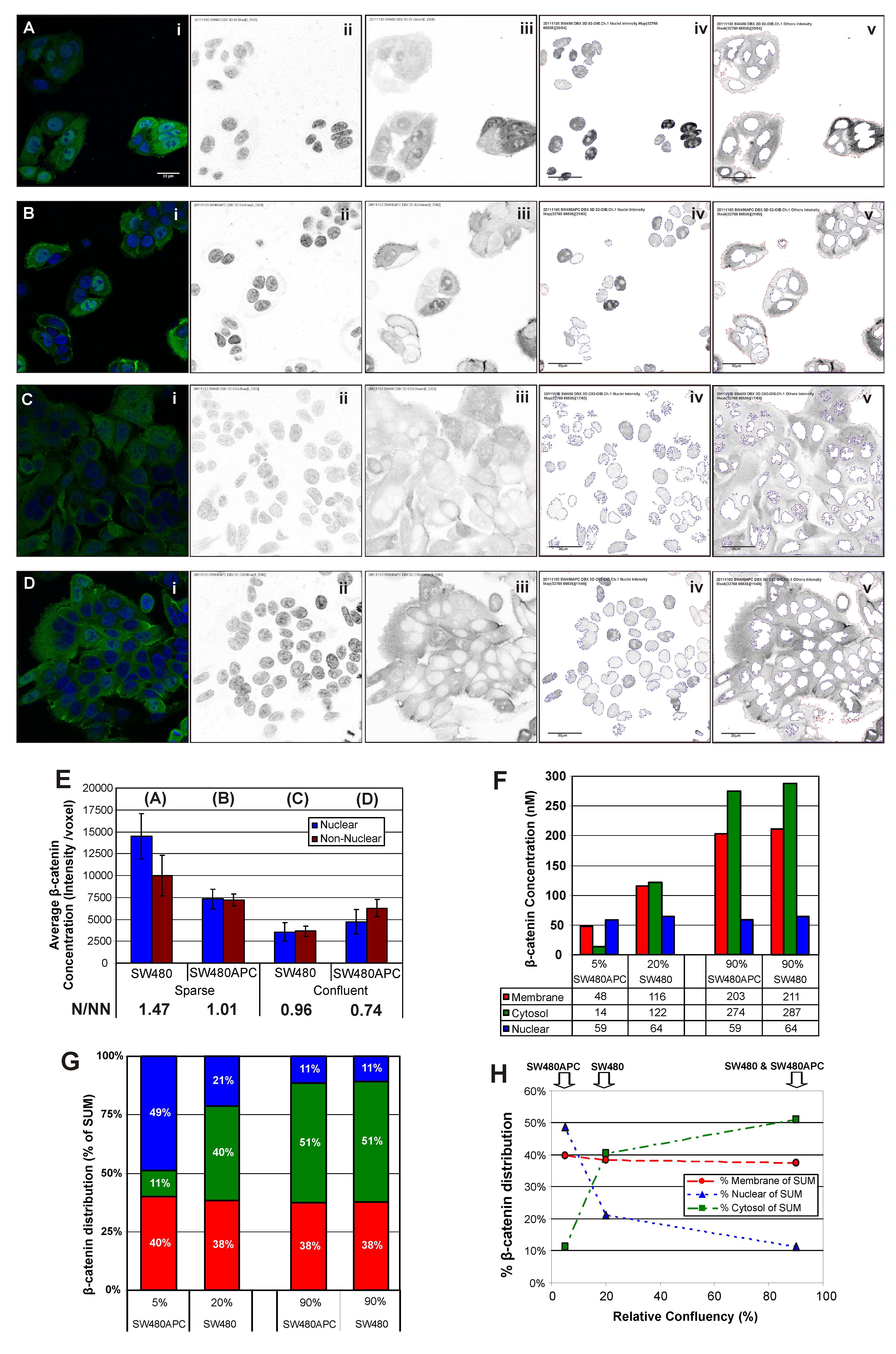

Supplement: Figure S10 — Dependency of β-catenin distribution in SW480 and SW480APC on confluency. 3D confocal imaging and compartment analysis at different confluency: (A) SW480 and (B) SW480APC at low confluency; (C) SW480 and (D) SW480APC at high confluency. Panels show the (i) overlay (DAPI in blue and β-catenin in green), (ii) DAPI, (iii) β-catenin, (iv) nuclear β-catenin intensity and (v) non-nuclear β-catenin intensity images. (E) Results of compartmental analysis in intensity per voxel shows higher nuclear∶non-nuclear β-catenin ratio (>1) for low confluency samples. Lower nuclear∶non-nuclear β-catenin ratio (<1) is observed for highly confluent samples. (F) Sub-cellular fractionation results for both cell-lines at difference confluency in β-catenin concentrations (nM). Increasing cytosolic and membrane β-catenin concentrations are observed with increasing confluency with the nuclear β-catenin levels remaining constant. (G and H) β-catenin compartment distribution (in % of compartment summation), nuclear β-catenin distribution decreases and the cytosolic β-catenin level increases with increasing confluency. The membrane levels remained constant. Note: panels ii–v are grayscaled for visual clarity. (TIF) [file pone.0031882.s010.tif]

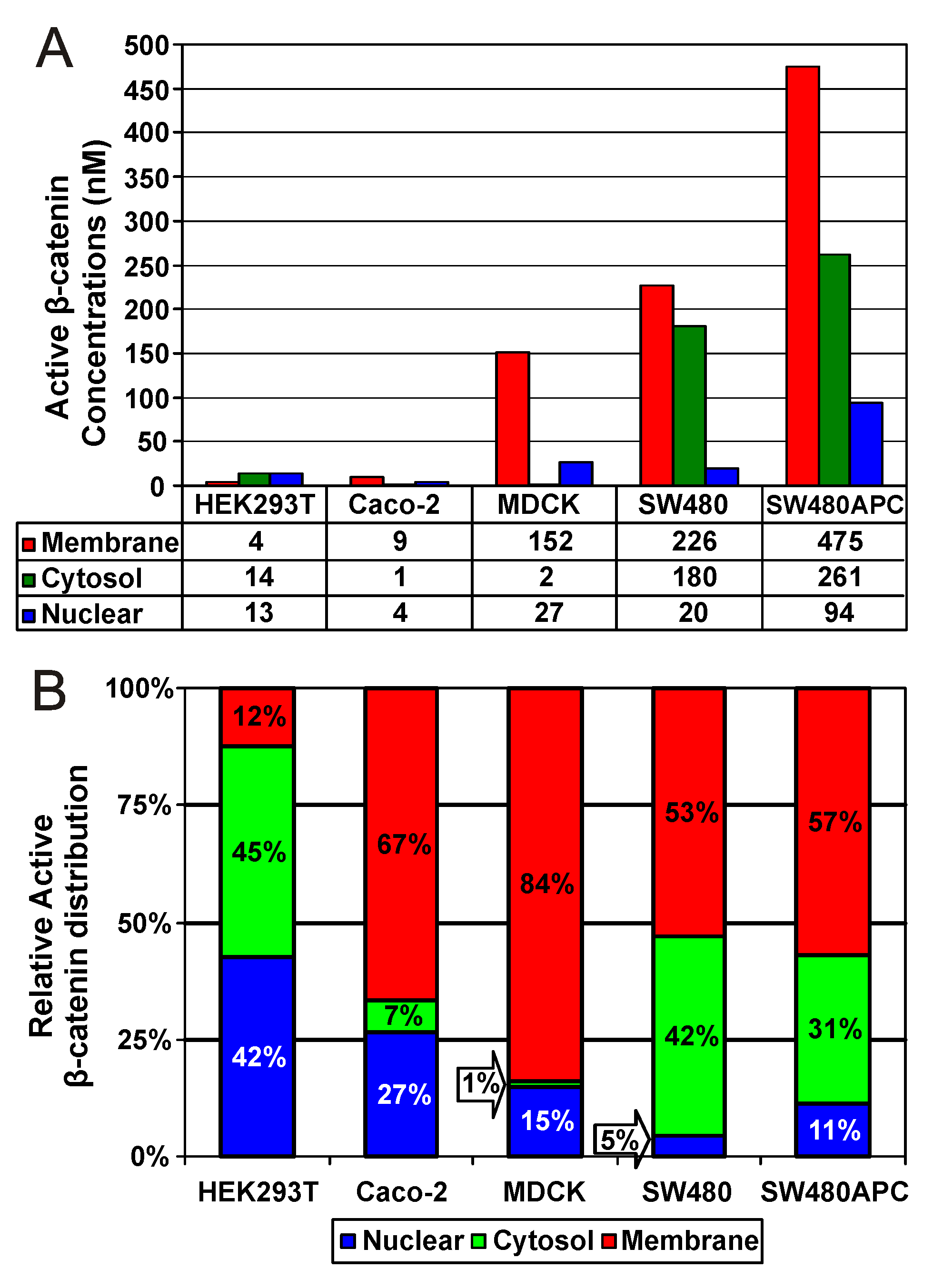

Supplement: Figure S11 — Sub-cellular distribution of “active” β-catenin in mammalian cells. (A) Compartment concentrations of “active” β-catenin in HEK293T, Caco-2, MDCK, SW480 and SW480APC cells. (B) Percentage distribution of “active” β-catenin for the five cell lines. (TIF) [file pone.0031882.s011.tif]

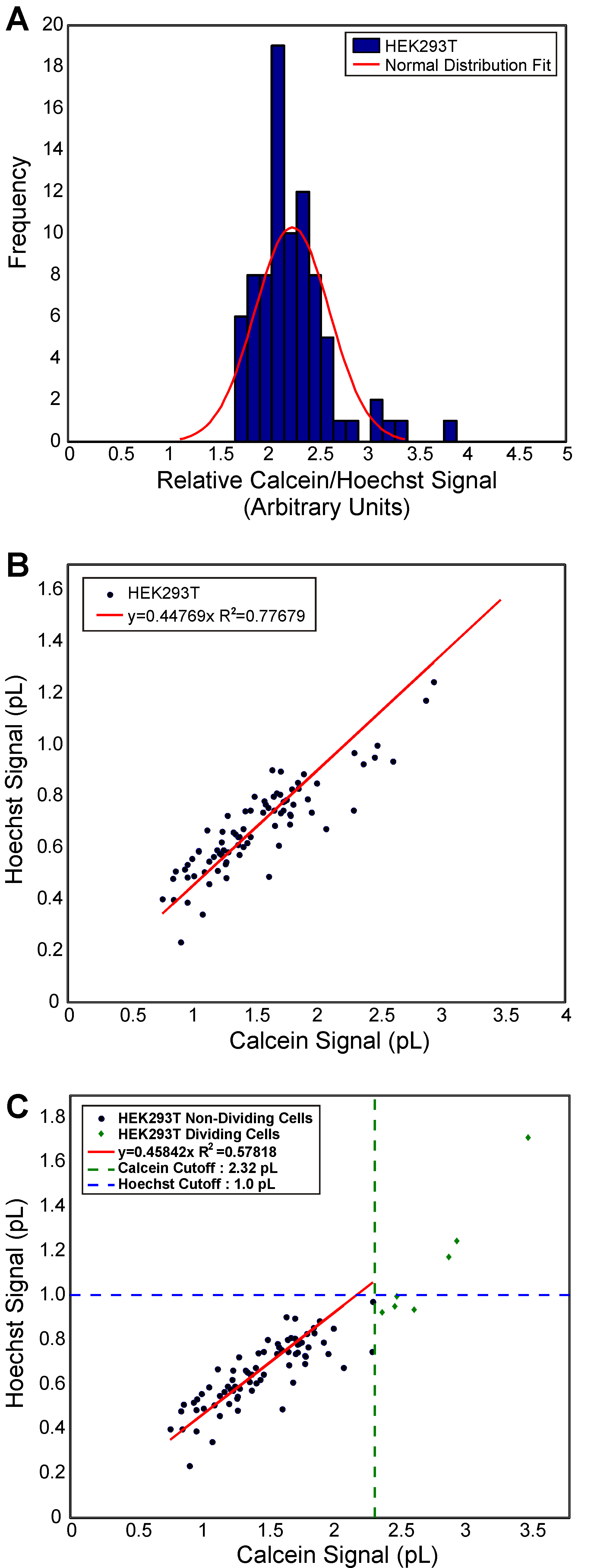

Supplement: Figure S12 — Cell population sizing and selection (HEK293T). Population statistics of HEK293T, showing the (A) histograms of normalised volume (Calcein AM/Hoechst); (B) Scatter plot of fluorescent marked volumes (Calcein AM vs. Hoechst) showing a good linear relationship; (C) Scatter plot of fluorescent marked volumes (Calcein AM vs. Hoechst) showing population isolation and the cut-off volumes applied. (TIF) [file pone.0031882.s012.tif]

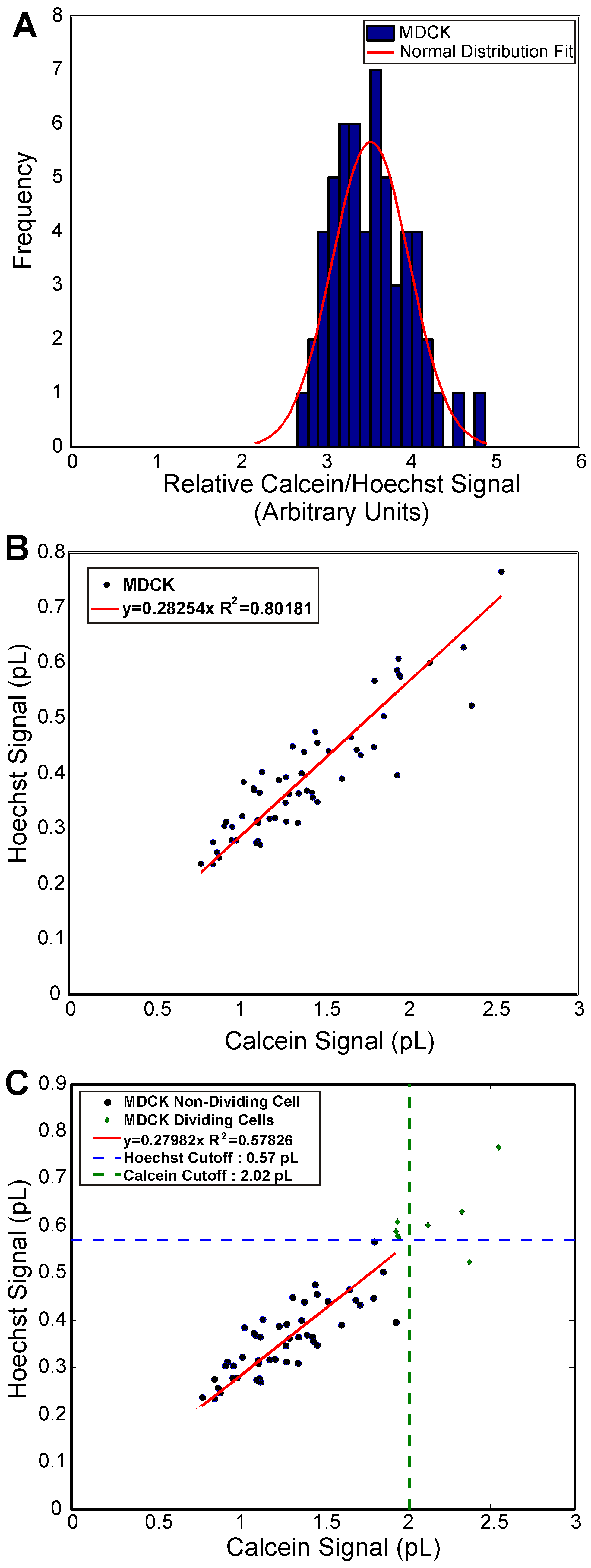

Supplement: Figure S13 — Cell population sizing and selection (MDCK). Population statistics of MDCK, showing the (A) histograms of normalised volume (Calcein AM/Hoechst); (B) Scatter plot of fluorescent marked volumes (Calcein AM vs. Hoechst) showing a good linear relationship; (C) Scatter plot of fluorescent marked volumes (Calcein AM vs. Hoechst) showing population isolation and the cut-off volumes applied. (TIF) [file pone.0031882.s013.tif]

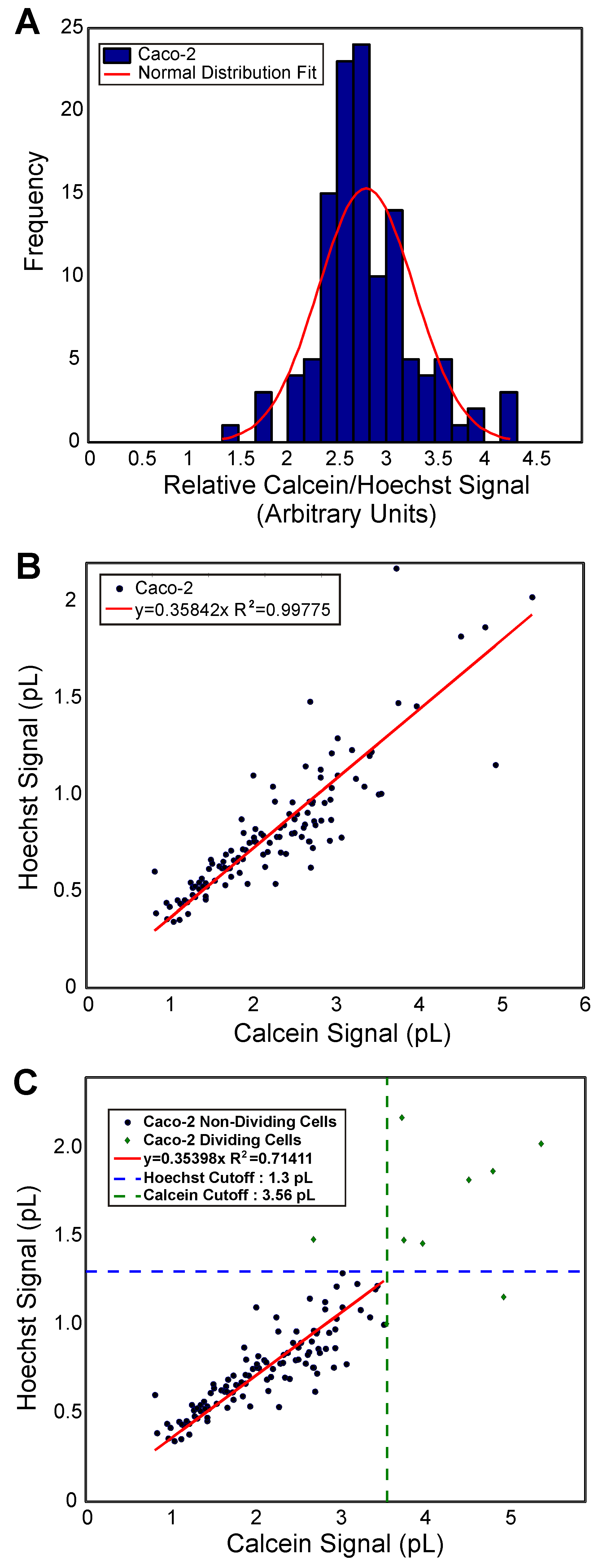

Supplement: Figure S14 — Cell population sizing and selection (Caco-2). Population statistics of Caco-2, showing the (A) histograms of normalised volume (Calcein AM/Hoechst); (B) Scatter plot of fluorescent marked volumes (Calcein AM vs. Hoechst) showing a good linear relationship; (C) Scatter plot of fluorescent marked volumes (Calcein AM vs. Hoechst) showing population isolation and the cut-off volumes applied. (TIF) [file pone.0031882.s014.tif]

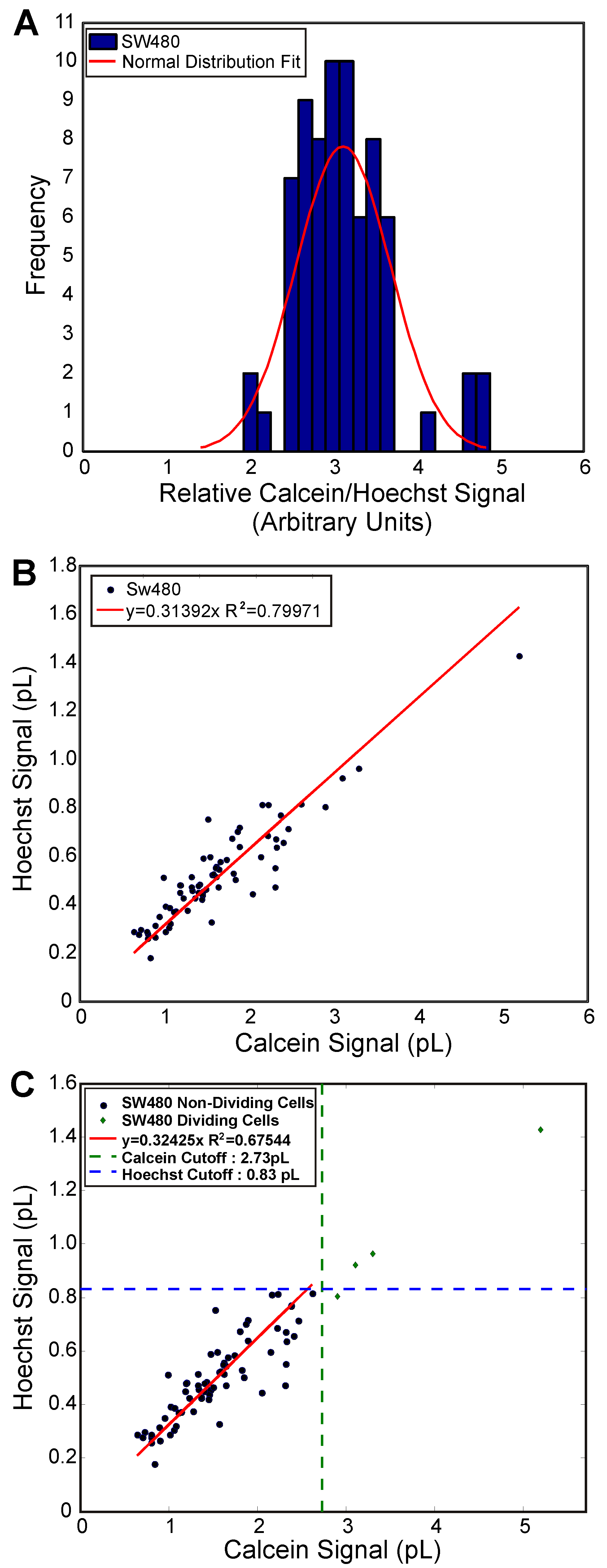

Supplement: Figure S15 — Cell population sizing and selection (SW480). Population statistics of SW480, showing the (A) histograms of normalised volume (Calcein AM/Hoechst); (B) Scatter plot of fluorescent marked volumes (Calcein AM vs. Hoechst) showing a good linear relationship; (C) Scatter plot of fluorescent marked volumes (Calcein AM vs. Hoechst) showing population isolation and the cut-off volumes applied. (TIF) [file pone.0031882.s015.tif]
