# Supplementary material for: Wnt Signalling Pathway Parameters for Mammalian Cells
Source: PLoS One. 2012 Feb 21;7(2):e31882. doi: 10.1371/journal.pone.0031882 (PMC3283727; doi:10.1371/journal.pone.0031882)
Supplement: Text S1 — Preparation and Purification of Recombinant Proteins. (DOC) [file pone.0031882.s016.doc]

**Preparation and Purification of Recombinant Proteins**

**Recombinant Full-length β-cateninEE**

A cDNA encoding full-length murine β-catenin (residues 1-782) with the epitope Glu-Glu (EE) tag and a 3 amino acid spacer appended to the C-terminus by PCR was subcloned into the baculovirus transfer vector, pBlueBac4 (Invitrogen, Carlsbad, CA, USA) and co-transfected into Sf9 cells with Bac-N-Blue baculovirus DNA (Invitrogen, Carlsbad, CA, USA). The EE epitope tag comprises 6 amino acids leading to a new C-terminal sequence of PGGEYMPME. The resultant recombinant baculoviruses were plaque purified and amplified as described previously . Sf9 cells were grown exponentially (to a density of 1.5 - 2.0 × 10^6^ cells/ml) and infected with the recombinant baculoviruses at between 2 and 10 multiplicity of infection. The Sf9 cells (between 5 × 10^9^ - 2 × 10^10^ cells) were harvested 3 days post infection and lysed by nitrogen cavitation (in 20mM Tris pH 7.4, 50mM NaCl containing 2 mM 2-mercaptoethanol (2-ME) and Complete^TM^ protease inhibitors at a pressure of 1500 psi for 20 minutes) and the lysate clarified by centrifugation (200,000*g* for 1 hour at 4^o^C) before the supernatant was filtered through a 0.22µm membrane.

β-cateninEE was purified by affinity chromatography using an antibody directed against the EE epitope-tag . Purified anti-EE monoclonal antibody (mAb) was immobilised on Protein G Sepharose Fast Flow (Amersham Biosciences) by covalent cross-linking (at 1-2 mg mAb/ml of gel). β-cateninEE Sf9 supernatant fluid was loaded onto a 175 x 6.6mm column of anti-EE mAb Protein G Sepharose equilibrated in 20mM Tris pH 7.4, 50mM NaCl containing 2 mM 2-ME, and washed with 10 column volumes of the same buffer at 2ml/min. β-cateninEE was competitively eluted with a 5 column volume linear gradient of 0-50 µg/ml EE-tag peptide (EYMPME**,** Auspep) in the same buffer. Recombinant β-cateninEE was further purified by anion exchange chromatography using a Poros 20 HQ column equilibrated with 20mM Tris pH 7.5 containing 2mM 2-ME with the elution carried out using a 30 column volume linear gradient from 0.2-0.7M NaCl at 2ml/min. Fractions were analysed using Coomassie-stained SDS-PAGE and the concentration of purified β-cateninEE was quantified by UV spectroscopy using a molar extinction coefficient of 62910 at 280nm as calculated from amino acid composition .

**Recombinant Full-length FLAG-GSK3β**

Recombinant full-length GSK3β was prepared using a cDNA encoding full-length human GSK3β with a FLAG tag added to create a new N-terminal sequence MDYKDDDDK followed by residue 2 of GSK3β. FLAG-tagged GSK3β (FLAG-GSK3β) cDNA was subcloned into pBlueBac4, co-transfected into Sf9 cells with Bac-N-Blue baculovirus DNA, and recombinant baculoviruses were plaque purified and amplified as described . Protein expression was carried out in Sf9 cells as described for β-cateninEE. Sf9 cells (~2-5 × 10^9^ cells) expressing FLAG-GSK3β were lysed in 20mM HEPES pH7.4 containing 150mM NaCl, 2mM 2-ME, 1% Triton X100 and Complete^TM^ protease inhibitors and the lysate clarified by centrifugation at 13,000*g* for 20 minutes at 4^o^C. FLAG-GSK3β was purified by affinity precipitation using the M2 anti-FLAG tag monoclonal antibody covalently coupled to agarose (Invitrogen, cat # 18300-012, Carlsbaad, CA, USA). The affinity precipitates were solubilised in SDS-PAGE sample buffer and the amount of FLAG-GSK3β in the precipitate estimated by Coomassie Blue stained SDS-PAGE using known amounts of BSA on the same gel as a protein standard.

**Recombinant H_6_-m Axin-HA**

Recombinant H_6_-m Axin-HA was prepared using a cDNA encoding murine Axin with a 3 residue space and a haemagluttinin (HA) tag, giving a new C-terminal sequence VEKVDGGPMYPYDVPDYA. The cDNA was sub-cloned into the pBlueBacHis2 vector so that it was in frame with a hexa-his tag and an enterokinase cleavage site such that the new N-terminal sequence was MPRGSHHHHHHGMASMTGGQQMGRDLYDDDDKDRWIRPRDLHAAMQSPKM where the last 6 residues are from Axin. The N-terminally His-tagged, C-terminally HA-tagged Axin (H_6_-m Axin-HA) cDNA was co-transfected into Sf9 cells with Bac-N-Blue baculovirus DNA, and recombinant baculoviruses were plaque purified and amplified as described . Sf9 cells (~2-5 × 10^9^ cells) expressing H_6_-m Axin-HA were lysed in phosphate-buffered saline (PBS) containing 2mM 2-ME, 1% sodium deoxycholate, 1% Triton X100 and Complete^TM^ protease inhibitors and the lysate clarified by centrifugation at 13,000*g* for 20 minutes at 4^o^C. H_6_-m Axin-HA was enriched by affinity precipitation using Ni-NTA-agarose (Qiagen, cat #31210, Hilden, Germany) or the cobalt-based TALON immobilized metal affinity resin (Clontech, cat #635501, Mountain View, CA, USA). The affinity precipitates were solubilised in SDS-PAGE sample buffer and the amount of H_6_-m Axin-HA in the precipitate was estimated by Coomassie Blue stained SDS-PAGE using known amounts of BSA on the same gel as a protein standard.

**Recombinant Full-length H_6_-APC-EE**

Recombinant full-length H_6_-APC-EE was prepared using a cDNA encoding full length human APC with a 3 residue spacer and an EE tag added to the C-terminus (at residue 2843), creating a new C-terminal sequence PGGEYMPME. The cDNA was sub-cloned into the pBlueBacHis vector so that it was in frame with a hexa-his tag and an enterokinase cleavage site such that the new N-terminal sequence was MPRGSHHHHHHGMASMTGGQQMGRDLYDDDDKDRWGSELHRGGGKKLVD followed by residues MAAASY from APC. The N-terminally His-tagged, C-terminally EE-tagged human APC (H_6_-APC-EE) cDNA was co-transfected into Sf9 cells with Bac-N-Blue baculovirus DNA, and recombinant baculoviruses were plaque purified and amplified as described . Sf9 cells (approximately 2-5 × 10^9^ cells) expressing H_6_-APC-EE were lysed in Tris-buffered saline (TBS) containing 1% Triton X100 and Complete^TM^ protease inhibitors and lysate clarified by centrifugation at 13,000*g* for 20 minutes at 4^o^C. H_6_-APC-EE was enriched by affinity precipitation using the anti-EE tag monoclonal antibody and Protein G Sepharose. Affinity precipitates were solubilised in SDS-PAGE sample buffer and the amount of H_6_-APC-EE in the precipitate was estimated by Coomassie Blue stained SDS-PAGE using known amounts of BSA on the same gel as a protein standard.

**Recombinant Intracellular Domain of E-cadherin**

Recombinant intracellular domain of E-cadherin was prepared using a cDNA encoding human E-cadherin intracellular domain (ICD) (residues 736-882 of full-length human E-cadherin) sub-cloned into the GST fusion vector, pGEX-4T-1 (Amersham Biosciences). Plasmids were transformed into the BL21 (DE3) strain of *E. coli* and grown to log phase and protein expression induced with 0.2nM IPTG for 4 h at 37 °C. Bacterial pellets obtained by centrifugation (4000*g* for 15 minutes at 4°C), Dounce homogenised in 20 mM HEPES pH 7.4 containing 20 mM NaCl, 5 mM EDTA, 0.1 mg/ml lysozyme and Complete^TM^ protease inhibitors (Roche Applied Science), incubated on ice for 15 minutes, and sonicated (4 × 30 sec pulses) with addition of 5 mM DTT, sarcosyl (2% w/v) and Triton X100 (1% w/v) was then added and lysate clarified by centrifugation (13,000 × *g* for 20 minutes at 40°C) and filtered through a 0.22μm filter. The supernatant was loaded onto a TBS equilibrated glutathione agarose column (16 × 70mm) (Sigma, cat#G3907 St Louis, MO, USA), washed with 10 columns volumes of TBS at 4ml/min. The bound proteins were eluted with 2 column volumes of 50mM Tris pH 8 containing 10mM reduced glutathione at 2ml/min. Recombinant GST-E-cadherin ICD was further purified by anion exchange chromatography using a Bio-Scale Q2 column (4.6 × 60mm, Bio-Rad) equilibrated with 20mM Tris pH7.5 containing 2mM 2-ME. Proteins were eluted over 50 column volumes with a 0.25-0.75M NaCl gradient at a flow rate of 2ml/min. Chromatographic fractions were analysed using Coomassie-stained SDS-PAGE. Concentrations of purified GST-E-cadherin ICD were quantified by UV spectroscopy using a molar extinction coefficient of 63260 at 280nm as calculated from the amino acid composition .

**Reference**
