# Supplementary material for: Wnt Signalling Pathway Parameters for Mammalian Cells
Source: PLoS One. 2012 Feb 21;7(2):e31882. doi: 10.1371/journal.pone.0031882 (PMC3283727; doi:10.1371/journal.pone.0031882)
Supplement: Text S2 — Protocol for confocal cell compartment volume measurements and analysis. (DOC) [file pone.0031882.s017.doc]

**Protocol for confocal cell compartment volume measurements and analysis**

**Preparation and Staining for Confocal Microscopy**

Sub-confluent cells were harvested using trypsin/EDTA, washed twice with phosphate buffered saline and reconstituted as a single cell suspension in GIBCO’s phenol red-free RPMI. An aliquot (~1 × 10^6^ cells/ml) was then mixed with Phenylarsine Oxide (10µM) in the same medium. A mixture of Calcein AM fluorescent dye (1µM), Hoechst 33342 nucleic acid stain (2µg/ml) and Vybrant DiI cell labelling solution (3.5µM) was then added to the mixture and incubated for 20 minutes at 37ºC, washed twice with phenol-red-free RPMI and resuspended in 1ml phenol-red-free RPMI. Labelled cells were kept on ice prior to imaging.

To minimise the movement of cells (motion artefacts due to liquid movement) during imaging, labelled cells were diluted 1 in 20 and embedded in agarose (type) mixture was in a Sykes Moore Chamber (Bellco Glass Inc., Vineland, NJ). The cells were found to be viable for approximately 1 hour after embedding in agarose. Stained, agarose-embedded cells were imaged using an Olympus FV1000 confocal microscope with an Olympus, Model Uplan-SApo, x60, NA=1.2 water immersion objective lens.

**3D Confocal Fluorescence Microscopy Image Stack Acquisition**

Immunofluorescent staining was detected with an Olympus FV1000 Spectral Confocal attachment to an Olympus IX-81 microscope. The cells were imaged using standard filter sets and laser lines to acquire single labelled images. Hoechst 33342, Calcein AM and Vybrant DiI fluorescence were excited with the 405nm, 488nm and 546nm laser lines, respectively and the emission wavelength were measured at wavelengths 405nm, 473nm and 559nm, respectively. The images were captured using Olympus FluoroView software (Version 1.7c).

Z-stacks were acquired from the base of the cells (at the cover slip) to beyond the top of the cells to encompass all the cells in the region of interest (ROI). The number of 2D images per z-stacks was dependent on the thickness of the cells being imaged and the step size. Previous studies have inferred 3D cell volumes from single section information, from partial stacks or even from cell diameter , however these approaches are less accurate when cells are not spherical or homogenous. In addition, 3D reconstruction from z-stacks may allow future measurements of volumes of specific cell compartments and allow quantitation of protein translocation.

Each individual 2D sectional image in the acquired z-stack was 512 x 512 pixels. The objective and zoom factor resulted in a pixel dimension of approximately 0.15-0.35µm. The step size was synchronised with the pixel size to give cubic voxel sizes of approximately 0.15-0.35µm^3^. The matching of optical section/axial thickness to the step z-plane size ensures optimal axial sampling. Cubic voxels reduce spherical aberration to ensure optimal and reliable volumetric measurement .

The output analogue signal, representing the fluorescence intensities detected, was digitized to 16 bits resolution at 65536 levels of grey level (2^16^ discrete levels of information). The image stack produced was saved as an Olympus Image Binary (OIB) image. For each ROI only non-overlapping, live cells were analysed. Live cells were detected by their ability to retain the calcein AM dye . Imaging emission and detection settings were adjusted to an optimal level, minimizing pixel saturation for each channel. The offset and gain of the system was also utilised to minimise background signal, thus removing the need for further background correction during image and data processing.

**Image Analysis and Estimation of Cell Compartment Volumes**

Pre-processing of the acquired images was conducted using Metamorph Premier (v7.6.3.0, MDS Analytical Tech.,). The selection of non-overlapping cells for analysis was carried out using Metamorph by inspection of the cross-sectional profiles and visualisation using 3D reconstruction. After cell selection, an independent image stack of the cell was extracted and saved as a Tagged Image File Format (TIFF) image stack for further processing. Figure S1 shows the general steps taken in the image stack analysis and selection of cells.

The z-stack images were processed in Matlab as described in Figure S2-S4. TIFF images were imported and processed using the *Image Processing Toolbox*. Once imported, image stacks were separated into the independent channel images, filtered to emphasize the borders and thresholded automatically using the *Otsu auto-threshold method* to create a binary image mask. Otsu’s method chooses the threshold level of the object of interest to minimize the intra-class variance of the black and white pixels. The 3D application in Metamorph Premier is also capable providing an estimate of the cellular volume. However, that method requires visual and manual determination of the object threshold levels using a slider bar. Using visual inspection to determine threshold levels is highly subjective which adversely affects the resulting volume and the results are difficult to reproduce. Consequently, Matlab’s ‘*Image Processing Toolbox*’ *Otsu auto-threshold method* was used to determine automatically thresholds. This provides an objective, consistent and reproducible method to determine the optimal threshold for cellular segmentation.

Based on the threshold binary image mask, the images were segmented to isolate the respective volumes occupied by the three fluorescent signal markers. Intuitively, the volume would be obtained from the sum of the marked pixel volumes for each section. However, the procedure can be influenced by the presence of debris captured in the same field of view as the cell of interest. In order to confirm that the cellular volumes calculated were based on 3D cellular objects, the z stacks were processed only when objects connected in 3D were extracted. These extracted object volumes were calculated and small fragments of debris isolated and removed by volume filtering. This analysis therefore reduces noise that may occur when only considering 2D sections independently and enabled a more accurate measurement of each 3D cellular object by taking into account spatial connectivity and relationship between each plane of the 3D stack. Disconnected and isolated fragments or debris will therefore not be falsely counted as part of the cellular object. The occupied volumes were filled internally and processed as follows: the three compartment volumes acquired were the nuclear (Hoechst), inner cytoplasm plus nuclear (Calcein AM) and the whole cell volumes (Vybrant DiI). Using these three compartment volumes, the volume distribution of the nuclear, inner-cytoplasm and membrane-outer-cytoplasm compartments could be calculated by exclusion using the procedure depicted in Figure S3. The nuclear and cytoplasm volume information was used to distinguish and isolate the resting and dividing populations. Volumetric data of the three compartments or the whole cell was then calculated from the population statistics for the resting cells.

**Cellular Compartment Volumes Analysis and Population Isolation**

Correlations of nuclear content with cellular volume of the acquired cell population were analysed. Firstly normalised ratios between viable cell volume (marked by Calcein AM) and nuclear volume (marked by Hoechst 33342) were calculated and fitted to a normal distribution. Secondly, scatter plots and empirical linear data fitting to a linear equation y=Ax were estimated for Hoechst versus Calcein AM volumes. These analyse ascertains the basis for cell population identification and isolation.

Two populations of cells were defined in this study, namely a resting (non-dividing) population and a dividing population. The criteria for the cut-off (i.e. *CO_calcein_* and *CO_hoechst_*) to distinguish the two populations (i.e. dividing vs. non-dividing cell populations) was empirically set at 1.5 standard deviations as shown in Equation 1 and Equation 2. Specifically, the non-dividing population is defined to be any cell with both viable cell volume (*V_calcein_*, marked by calcein AM) and nuclear volume (*V_hoechst_*, marked by Hoechst 33342) that was less than 1.5 standard deviations greater than the respective sample mean (Equation 3). The dividing population was defined to be the remaining cell population (Equation 4).

$$CO_{calcein}=1.5\times\sigma_{calcein}\times V_{calcein}$$

Equation 1. Cut-off volume criteria for calcein signal

$$CO_{hoechst}=1.5\times\sigma_{hoechst}\times V_{hoechst}$$

Equation 2. Cut-off volume criteria for hoechst signal

$$P_{non-dividing}=(V_{hoechst}<CO_{hoechst})\text{ and }(V_{calcein}<CO_{calcein})$$

Equation 3. Criteria for distinction of non-dividing cell population

$$P_{dividing}=(V_{hoechst}\geq CO_{hoechst})\text{ and }(V_{calcein}\geq CO_{calcein})$$

Equation 4. Criteria for distinction of dividing cell population

The non-dividing (resting) cell population volume information was used in this section to calculate the compartment volumes and proportion of the cell volume accounted for by each compartment in the five cell lines. The marked volume data are used to calculate the compartment volumes as per described in Figure S3, Step 5.In this study, Hoechst 33342 marked volume is directly used as the nuclear volume and the whole cell volume is taken to be that of the Vybrant DiI marked volume.

Three compartments were defined in this study, namely nuclear, inner cytoplasm and membrane-outer cytoplasm. The membrane-outer cytoplasm compartment represents the membrane and the thin cytoplasm region just under the membrane. This definition was made so as to take into consideration that in the current system, the size of the smallest sampled volume element (voxel) was limited by the spatial resolution during image acquisition which in this study was about 0.3-0.4µm. The thickness of the plasma membrane (about 9.2 to 10nm ) is therefore much smaller than the current limits of optical resolution of the microscope. Therefore the membrane-outer cytoplasm compartment includes both the membrane and the layer of cytoplasm immediately under the membrane. However, this did not consider axial distortion and other factors which further imply that the boundary pixels might only be partially representative of the precise boundary.

For each cell line, the compartment volumes and relative distribution was calculated from the marked volumes as well as the relative compartment contributions with respect to the nuclei volume, the approximate volume ratio of *Nuclei:Cytosol:Membrane-Outer* Cytoplasm (*N:C:M Ratio*, with the nuclei volume as reference) was calculated.

**Reference**

1. Prakash YS, Smithson KG, Sieck GC (1994) Application of the Cavalieri Principle in Volume Estimation Using Laser Confocal Microscopy. NeuroImage 1: 325-333.

2. Sieck GC, Mantilla CB, Prakash YS (1999) Volume measurements in confocal microscopy. Methods in enzymology: 296-315.

3. Metamorph (2009) Metamorph Premier. 7.6.3.0 ed: MDS Analytical Tech.

4. Mathworks (2007) MATLAB. version. 7.4.0.287 (R2007a) ed: The Mathworks, Inc.

5. Otsu N (1979) A threshold selection method from gray-level histograms. IEEE Transactions on Systems, Man and Cybernetics 9: 62-66.

6. Schneiter R, Brügger B, Sandhoff R, Zellnig G, Leber A, et al. (1999) Electrospray Ionization Tandem Mass Spectrometry (Esi-Ms/Ms) Analysis of the Lipid Molecular Species Composition of Yeast Subcellular Membranes Reveals Acyl Chain-Based Sorting/Remodeling of Distinct Molecular Species En Route to the Plasma Membrane. The Journal of Cell Biology 146: 741-754.

7. Hine R (2005) The Facts on File dictionary of biology: Infobase Publishing, 2005.
