# Supplementary material for: Wnt Signalling Pathway Parameters for Mammalian Cells
Source: PLoS One. 2012 Feb 21;7(2):e31882. doi: 10.1371/journal.pone.0031882 (PMC3283727; doi:10.1371/journal.pone.0031882)
Supplement: Text S3 — Measurement Protocol for Protein Concentrations in Mammalian Cells. (DOC) [file pone.0031882.s018.doc]

**Text S3: Measurement Protocol for Protein Concentrations in Mammalian Cells**

**Preparation of Whole Cell Lysate**

Cell cultures of the five cell lines (MDCK, HEK293T, SW480, SW480APC and SW620) were harvested when confluent by scraping into ice-cold PBS. Cells pellets were washed once in 5mL PBS and pelleted by centrifugation at 1500 rpm for 5 minutes at 4°C. Cells were lysed using 500µl of SDS lysis buffer (2% SDS in PBS containing a cocktail of Protease Inhibitors (Complete^TM^, Boehringer Mannheim). The lysate was incubated on ice for 10 mins, centrifuged at 543,000*g* for 1 hour at 4^o^C (Beckman TL100 rotor and ultracentrifuge) to remove DNA and insoluble material and supernatant whole cell lysate (WCL) was stored at -70°C. The Bicinchoninic Acid (BCA) Assay was used to determine the total amount of protein in the lysates.

**Quantitative Western Blots** **and Estimation of Total Protein per Cell**

Based on total protein concentrations calculated from the BCA assays, aliquots of whole cell lysates (WCL) corresponding to known amounts of total protein per lane were prepared for each cell line. Known amounts of the corresponding recombinant protein were loaded in parallel lanes on the gel to form a protein standard curve. Aliquots of whole cell lysates were separated by SDS-PAGE using 3-8% Tris-Acetate (for APC) or 4-12% Bis-Tris NuPAGE gels and Tris-Acetate or MOPS buffer according to the manufacturer’s recommendations (Invitrogen) then transferred onto nitrocellulose membrane using NuPAGE Transfer Buffer (Invitrogen) for 16 hours at 25V. Immunoblotting was carried out as recommended in the Western Blot Analysis Protocol in the Odyssey Infrared Imaging System Application Protocols manual (LI-COR Biosciences). Each gel compared lysates from the 5 cell lines and the four recombinant protein standards (Figure S5). Amounts of protein in WCLs loaded were: 20µg and 50µg when immunoblotting for β-catenin, 20µg and 50µg for E-cadherin, 50µg to 200µg for APC, 100µg to 300µg for Axin and 50µg to 100µg for GSK3β. Recombinant protein ranges were: 1ng to 10ng for β-catenin, 1ng to 10ng for E-cadherin, 2ng to 100ng for APC, 10ng to 500ng Axin and 1ng to 50ng for GSK3β. The levels of proteins were measured using LICOR Odyssey^TM^ Infrared Imaging System (LI-COR, Inc., Lincoln, NE, USA). A standard curve was constructed from the Integrated Intensity (*II*) readout for the recombinant proteins plotted against the corresponding amount of protein loaded and the amount of protein in the loaded volume of lysate was calculated using the specific standard curve. Subsequently the amount of protein per µg of total protein in each cell line was calculated based on the steps described (Steps A-C in Figure S6).

The total protein per cell was calculated by counting cells prior to lysis and BCA assay. The five cell lines were detached using trypsin/EDTA and washed twice with HT-PBS. 20% of the sample was used to count the total number of cells using a hemocytometer. The rest of the sample was analysed by BCA assay. Three replicates were counted per each cell line and the experiment was repeated twice. The cell number in the original sample was calculated based on Equation 1. The total protein in the original lysate sample was calculated using the BCA Assay. For the BCA Assay, the cells were lysed in 150µl of 2% SDS and protein assay conducted as per the manufacturer’s instructions. The total protein in the original lysate was calculated based on Equation 2. Taken together, the estimation for the total protein per cell was calculated based on Equation 3 for each cell line.

$Cnt_{TL}=\frac{Cnt_{m}\times V_{S}}{f_{L}}$

Equation 1. Total cell count. *Cnt_TL_* is the total cell count of the lysate, *Cnt_m_* is the mean cell count per ml, *V_S_* is sample volume (ml) and *f_L_* is fraction of original lysate volume accounted for by the cell count assay.

$M_{TL}=\frac{C\times V_{A}}{f_{L}}$

Equation 2. Mass of total protein: *M_TL_* is the mass of total protein in the lysate (mg), *C* is the protein concentration (mg/ml) measured using protein assay, *V_A_* is aliquot volume analysed (ml) and *f_L_* is fraction of original lysate volume accounted for by the protein assay.

$M_{TC}=\frac{M_{TL}}{Cnt_{TL}}$

Equation 3. Mass of total protein per cell: *M_TC_* is the mass of total protein per cell (ng/cell), *M_TL_* is the mass of total protein in the cell lysate (ng) calculated in Equation 2 and *Cnt_TL_* is the total cell count of the lysate calculated in Equation 1.

**Cellular Protein Concentration**

Quantitative immunoblot analysis uses a standard curve of known protein mass to estimate the amount of a protein of interest in each cell line with respect to amount of total protein (i.e. mass of specific protein in each µg of total protein). The cell count combined with a total protein assay was used to calculate the mass of total protein per cell and analysis using confocal microscopy was used to derive the cell volume for each of the five cell lines (as described earlier). The whole cell volume data for the resting (non-dividing) cellular populations were employed in this study. Integrating the information together, the concentration (in nanomolar) of the key Wnt proteins in each cell line was calculated as described in the steps shown in Figure S6. Specifically the amount of each protein per cell was determined by multiplying the amount of protein per µg of total protein (D) by the total protein mass per cell (E). This protein mass was then converted to moles (G) by dividing by the relative molecular weight of the protein. The concentration in molar of protein per cell (I) was then calculated by dividing number of moles of protein per cell (G) by the measured cellular volume corresponding to the cell line of interest (H). The molar concentration was finally converted to nanomolar (J). The calculations for A-D were the average of up to 4 independent immunoblot experiments for each of the five proteins, for each of the five cell lines.

**Sub-Cellular Fractionation of mammalian cells**

A sub-cellular fractionation protocol adapted and modified from that edited by Abcam (Abcam plc, Cambridge, MA), provided by Dr. Richard Pattern (Tufts-New England Medical Centre, Molecular Cardiology Research Centre, Boston, MA ) was employed for isolating the proteins from the different compartments from the whole cell. The compartments fractions were the Cytosolic (CF), Membrane (MF) and Nuclear (NF) fractions.

Cultured cells were plated and grown in large dishes (22 cm x 2 for each cell line) until confluent. When harvesting, cell counting was conducted on one of the dishes while for the other, the media was removed, cells were washed with PBS before 2mL of Sub-cellular Fraction Buffer (**SFB**, 250 mM Sucrose, 20 mM HEPES pH7.4, 10mM KCl, 1.5mM MgCl_2_, 1mM EDTA and 1mM EGTA without protein inhibitors) was added. The cells were scraped off and the cell suspension was transferred into a dounce homogenizer (7 ml, type B with a tight piston, Kontes Glass Co., Vineland, NJ). Homogenization was carried out for 10 minutes on ice; the suspension was then transferred into a 10 mL tube and incubated on ice for 20 minutes. The total volume of the mixture was noted at this point. 200μl of the mixture was extracted as the whole cell lysate (WCL fraction) and kept on ice.

The nuclear fraction (pellet) (NF) was collected by centrifuging at 720G (3000rpm) for 5 minutes at 4˚C and kept on ice. 2ml of the supernatant fluid (membrane and cytosolic fractions) was removed and transferred into an ultracentrifuge tube (Beckman Coulter 343778, 1mL per tube). The membrane fraction (pellet) (MF) was centrifuged out at 100,000G (40,000rpm) for 1 hour on a Beckman Coulter Optima TLX-120 Ultracentrifuge (Beckman Coulter, Inc., Brea, CA) and kept on ice. The resulting supernatant fluid was the cytosolic (CF) fraction. The remaining pellet was resuspended (using a 25G needle) in 400μl of SFB and re-centrifuged for 45 minutes at 100,000G (40,000rpm) on the Ultracentrifuge. The pellet was the membrane (MF) fraction which was resuspend in 2ml of lysis buffer. The lysis buffer (LB) is made up of 10% glycerol, 0.1% SDS in deoxycolate lysis buffer (20mM HEPES, 5mM EDTA, 150mM NaCl, 1% TritonX-100, 1% Na Deoxycholate and one Protease Inhibitor tablet).

The nuclear pellet was resuspended in 500μl of SFB (using a 25G needle 10 times) and centrifuged at 720G (3000rpm) for 5 minutes at 4˚C. The supernatant was discarded and process repeated with a 10 minutes centrifugation at 4˚C. The resulting pellet was resuspended in 2mL of LB (same as that of the membrane fraction). This mixture was mixed well and sonicated on ice for 30 seconds. For all fractions, 200μl was extracted, 23% 4x sample buffer and 9% of 0.5M DTT was added and boiled for 5 minutes at 95˚C (NF boiled for 30 minutes).

**Estimation of Compartment β-catenin Concentrations**

For each cell line, aliquots of fraction lysates were separated by SDS-PAGE using 4-12% Bis-Tris NuPAGE gels and MOPS buffer for all fractions (NF, MF, CF and WCL). Known amounts of the corresponding recombinant β-catenin protein were loaded in parallel lanes on each gel to form the standard curve. Typical lane configurations were shown in Figure S7. The proteins were transferred onto nitrocellulose membrane using NuPAGE Transfer Buffer (Invitrogen) for 4 hours at 90V and immunoblotting carried out as per recommended (discussed earlier) for either total or “active” β-catenin. β-tubulin was used as a cytoplasmic marker (fractionation validation). The levels of proteins were measured using LICOR Odyssey^TM^ Infrared Imaging System (LI-COR, Inc., Lincoln, NE, USA).

In order to relate recombinant β-catenin standards intensity of “active” β-catenin to that of total β-catenin, an independent western blot with two identical set of recombinant β-catenin standard curve lanes where one set was probed for total β-catenin (i.e. anti-β-catenin monoclonal antibodies (Transduction Laboratories, mouse 610153, BD Biosciences, San Jose, CA)) while the other was probed with “active” β-catenin using anti-Active-β-catenin (Millipore, mouse clone 8E7, cat#05-665, Temacula, CA) antibodies.

Integrating this mass per lysate volume information with total cell count per dish, volume of lysate loaded and the cellular volume per cell (discussed earlier), the concentrations of β-catenin (Total or active in nanomolar) per cell for each cell line were calculated based on the steps described (Steps E-L in Figure S9).

In this experiment, the total amount of β-catenin or “active” β-catenin in the experiment was determined by scaling up the amount of protein per lane (E) off the Western blot to the amount in the loading sample (F), and subsequently in the whole cell mixture (G, volume from the homogenisation). Using cell count from a duplicate dish, the amount of protein per cell was calculated (H). This mass was converted to moles (K) by dividing by the relative molecular weight of β-catenin and measured cellular volume (J) and converted to nanomolar (L).

**Dependency of SW480 and SW480APC β-catenin compartment concentration distribution on cell culture conditions**

It was noted during sub-cellular fractionation experiments that the compartment distribution of β-catenin in SW480 and SW480APC cells was dependent on the density of the cells. This dependency has a significant effect on the spatial localisation of β-catenin in these two cell lines. 3D confocal imaging and quantification analysis was employed to measure the relative spatial compartment concentrations of β-catenin (in intensity per voxel) in these two cell lines at different level of confluency. The details of this 3D quantification technique are described in a manuscript currently in preparation.

In this experiment, SW480 and SW480APC cells were grown on glass slides to low (5-20%) and high (>90%) confluency levels respectively for each cell line. The cells were immuno-stained with anti-β-catenin (BD Transduction Laboratories, BD Biosciences, San Jose, CA, cat#610153), marking the endogenous β-catenin. The nuclei of the cells were marked using the fluorescent stain 4, 6-diamidino-2-phenyl indole Nucleic Acid Stain (DAPI, cat# D1306 Molecular Probes Inc, Eugene, OR, Invitrogen). The glass slides of stained cell cultures were mounted onto Sykes Moore Chambers (Bellco Glass Inc., Vineland, NJ). 3D image stacks of the stained cells were acquired using the Olympus FV1000 confocal microscope under a 60x water immersion lens and processed by Metamorph Premier image processing software. Image compartmental analysis was performed in Matlab to obtain the resultant β-catenin calculations for the nucleus (marked by the DAPI) and non-nucleus compartments. More than 3 image stacks were quantified for each cell line and confluency. Resulting confocal and analysis images were tabulated in Figure S10A-E and correlated with that obtained from sub-cellular fractionation experiments.

At low confluency, β-catenin levels were higher in the nucleus than the non-nuclear compartment (i.e. Nucleus/Non-Nucleus Ratio > 1), particularly for SW480 cells. At high confluency, both cell lines exhibited a Nucleus/Non-Nucleus Ratio of less than one. Corresponding sub-cellular fractionation results (Figure S10 F-H) indicated that with decreasing confluency, the nuclear β-catenin distribution decreases while cytosol β-catenin distribution increases. Membrane β-catenin distribution remained constant.

**Reference:**

1. Smith PK, Krohn RI, Hermanson GT, Mallia AK, Gartner FH, et al. (1985) Measurement of protein using bicinchoninic acid. Analytical Biochemistry 150: 76-85.
